# Exosome‐Mediated Lectin Pathway and Resistin‐MIF‐AA Metabolism Axis Drive Immune Dysfunction in Immune Thrombocytopenia

**DOI:** 10.1002/advs.202412378

**Published:** 2025-01-10

**Authors:** Jin Li, Xiaoqian Wang, Yaoyao Chen, Xianlei Sun, Liyan Fu, Qingxuan Xin, Huilin Zhang, Bo Qin, Nannan Sun, Yingmei Li, Yan Xu, Hui Yang, Dawei Huo, Yong Dong, Shuya Wang, Mengyun Zhao, Quande Lin, Fang Wang, Baohong Yue, Yanxia Gao, Yong Jiang, Rongqun Guo

**Affiliations:** ^1^ Translational Medical Center The First Affiliated Hospital of Zhengzhou University Zhengzhou Henan 450001 China; ^2^ Department of Hematology The First Affiliated Hospital of Zhengzhou University Zhengzhou Henan 450001 China; ^3^ Department of Laboratory Medicine The First Affiliated Hospital of Zhengzhou University Zhengzhou Henan 450001 China; ^4^ Basic Medical Research Center Academy of Medical Sciences Zhengzhou University Zhengzhou Henan 450052 China; ^5^ Department of Laboratory Medicine The First Affiliated Hospital of Henan University of Chinese Medicine Zhengzhou Henan 450046 China; ^6^ Translational Cancer Research Center Peking University First Hospital Beijing 100034 China; ^7^ Bone Marrow Transplantation Center of The First Affiliated Hospital & Liangzhu Laboratory Zhejiang University School of Medicine Zhejiang University Hang Zhou Zhejiang 311100 China; ^8^ Department of Immunology School of Basic Medical Sciences Chengdu Medical College Chengdu Sichuan 610500 China; ^9^ Department of Blood Transfusion The First Affiliated Hospital of Zhengzhou University Zhengzhou Henan 450001 China; ^10^ Department of Hematology The Affiliated Cancer Hospital of Zhengzhou University & Henan Cancer Hospital Zhengzhou Henan 450000 China; ^11^ Department of Emergency Medicine The First Affiliated Hospital of Zhengzhou University Zhengzhou Henan 450001 China; ^12^ Henan International Joint Laboratory of Infection and Immunity The First Affiliated Hospital Zhengzhou University Zhengzhou Henan 450001 China; ^13^ Henan Key Laboratory of Critical Care Medicine Department of Emergency Medicine The First Affiliated Hospital Zhengzhou University Zhengzhou Henan 450001 China; ^14^ Institute of Infection and Immunity Henan Academy of Innovations in Medical Science Zhengzhou Henan 451163 China

**Keywords:** arachidonic acid metabolism, exosome, immune thrombocytopenia, MIF, resistin

## Abstract

Immune thrombocytopenia (ITP) is an autoimmune disorder characterized by reduced platelet levels and heightened susceptibility to bleeding resulting from augmented autologous platelet destruction and diminished thrombopoiesis. Although antibody‐mediated autoimmune reactions are widely recognized as primary factors, the precise etiological agents that trigger ITP remain unidentified. The pathogenesis of ITP remains unclear owing to the absence of comprehensive high‐throughput data, except for the belated emergence of autoreactive antibodies. In this study, using flow cytometry (FCM), proteomics, and single‐cell RNA sequencing of samples from patients with ITP, it is shown that exosome‐mediated lectin complement pathway is involved in the pathogenesis of ITP, which triggers and enlarges the complement activation cascade without effective regulation because of downregulated CD55. The activated complement system enhances the immune response and resistin and further Macrophage Migration Inhibitory Factor (MIF) triggers several proinflammatory signaling pathways, which contribute to the survival of hyperactivated immune cells and dysfunctional arachidonic acid (AA) metabolism. The resistin and MIF are also identified as potential contributors to resistance to glucocorticoid therapy. Taken together, the findings indicate that the lectin pathway of the complement system, resistin, MIF, and AA metabolism may serve as promising targets for ITP treatment, offering novel perspectives on potential therapeutic interventions.

## Graphical abstract

Immune thrombocytopenia (ITP) is an autoimmune disorder marked by reduced platelet levels and heightened susceptibility to bleeding resulting from augmented autologous platelet destruction and diminished thrombopoiesis. It is found that the lectin pathway of the complement system, resistin, MIF, and AA metabolism may serve as promising targets for treatment of ITP, offering novel perspectives on potential therapeutic interventions.

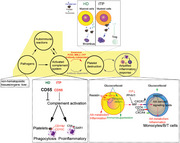

## 1 Introduction

Immune thrombocytopenia (ITP) is a complex autoimmune disorder characterized by a variable etiology, leading to bleeding and reduced platelet counts due to platelet destruction. The combination of high clearance and low platelet production contributes to the thrombocytopenia observed in ITP, with platelet destruction mediated by the mononuclear phagocytic system being considered a central event. A thorough understanding of the pathophysiology of ITP is crucial to determine appropriate treatment strategies. Autoimmune platelet destruction has also been observed in various other conditions, including infections (hepatitis C virus and human immunodeficiency virus), systemic lupus erythematosus (SLE), and multiple sclerosis.

ITP, a disease characterized by isolated thrombocytopenia, may present challenges for the early detection and identification of specific trigger factors. Autoantibodies targeting glycoproteins (GP) IIb/IIIa, Ib/IX, and V have been identified in many patients with ITP, leading to platelet clearance through the classical antibody‐mediated pathway. Treatment with anti‐D antibodies has shown efficacy, potentially attributable to mononuclear phagocyte system (MPS) blockade.^[^ ^1^ ^]^ Platelet autoantibodies are undetectable in ≈40% of patients with ITP, and targeting B cells and autoantibodies has proven ineffective.^[^ ^2^ ^]^ The lectin‐carbohydrate‐mediated Fc‐independent pathway, as a parallel pathway, contributes to platelet destruction because of the loss of sialic acid.^[^ ^3^ ^]^ Platelet surface exposure of glycoside residues due to sialic acid loss forces the removal of platelets through hepatic Ashwell–Morell receptors.^[^ ^4^ ^]^ Additionally, the upregulation of α1,6‐fucose and α‐mannose levels in platelets is a consequence of sialic acid loss.

Numerous additional factors, such as the dysfunction of regulatory T cells and dendritic cells (DC), CD8^+^ T cell‐mediated cytotoxicity, and impaired megakaryocyte function, play a role in the pathophysiology of ITP.^[^ ^5^ ^]^ Whole exome sequencing (WES) has revealed several deleterious variants, including IFNA17 (related to type I interferon signaling), ESS2, SMAD2 (associated with TGFβ/Smads signaling), CD83 (involved in antigen presentation regulation), IFNLR1 (linked to type III interferon signaling), and REL (related to nuclear factor [NF]‐κB signaling), in children with chronic ITP.^[^ ^6^ ^]^ Various therapeutic options, including glucocorticoids (GC), thrombopoietin receptor agonists, and intravenous immunoglobulin, have limitations, such as suboptimal efficacy, adverse side effects, and high cost.

The bone marrow (BM) niche is a spongy tissue responsible for platelet maturation and production, as well as antibody production,^[^ ^7^ ^]^ so exploring the pathogenesis of ITP may be more valuable in BM than in peripheral blood (PB). Therefore, we conducted a series of experiments using BM samples to elucidate the potential pathophysiology of ITP. Initially, a proteomic analysis was performed on BM plasma and BM plasma‐derived exosomes (EXOs), revealing the involvement of the exosome‐mediated lectin pathway of the complement system in platelet destruction. Subsequently, scRNA‐seq and FCM analysis identified CD55, C5AR1, resistin, and MIF as key factors that could serve as potential targets for ITP treatment. Additionally, our investigation revealed the role of resistin, revealing that it upregulates MIF and downregulates TSC22D3, thereby enhancing inflammatory responses and resistance to GC therapy. Furthermore, MIF has been identified as a key driver of the metabolic reprogramming of AA, potentially exacerbating the pathogenesis of ITP. In summary, our study provides evidence that the exosome‐mediated lectin pathway within the complement system is a potential contributor to ITP pathophysiology. Gene regulation networks of dysfunctional immune responses in patients with ITP were constructed emphasizing the targeting of the resistin‐MIF axis and dysfunctional AA metabolism as potential avenues for the development of ITP treatment options.

### 1.1 Exosomes from Patients with ITP Enhance Phagocytosis of MPS and Contribute to Platelet Destruction

**Figure 1:**
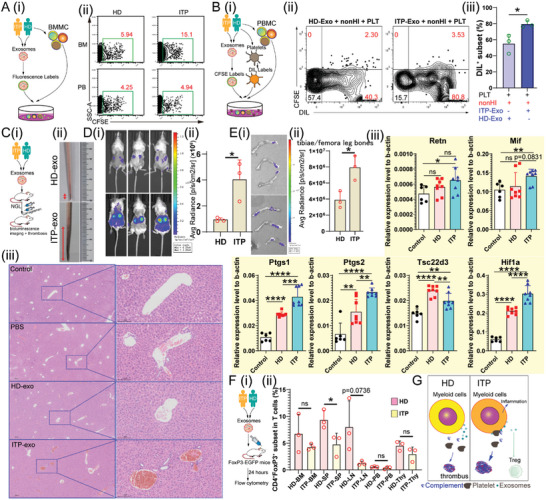
Identification of exosomes derived from patients with ITP that are linked to dysfunctional immune regulation. A). Experimental methodology for investigating variance in exosome interactions between patients with ITP and healthy donors with BM mononuclear cells (i). (ii) Representative flow cytometry plots illustrating the fluorescence intensity of CFSE after exosome treatment. B). Experimental methodology for examining the variances in exosome interactions between ITP patients and healthy donors with platelets and BM mononuclear cells (i). Representative proportions of CFSE‐labeled and DIL‐labeled CD14^+^ monocytes (ii). Quantification of DIL‐labeled cells as a percentage of CD14^+^ cells from ITP‐exosome (n = 3) and HD‐exosome treatments (n = 3) (iii). *P* value significance represented by *, <0.05; **, <0.01; ***, <0.001; ****, <0.0001. C). Experimental methodology for examining differences in carrageenan‐induced tail thrombosis (CTT) after treatment with exosomes from patients with ITP and healthy donors (i). Representative images of macrothrombi in the caudal vein 48 h after carrageenan administration (ii). *P* value significance represented by *, <0.05; **, <0.01; ***, <0.001; ****, <0.0001. Hematoxylin and eosin (H&E) staining of livers from different groups (iii). D). Representative images of the CTT NGL mice exhibiting bioluminescence signals (i). Box plots illustrating bioluminescent signals in CTT NGL mice treated with exosomes from patients with ITP (n = 3) and healthy donors (n = 3) (ii). *P* value significance represented by *, <0.05; **, <0.01; ***, <0.001; ****, <0.0001. E). Representative images of the tibiae and femoral leg bones from CTT NGL mice showing bioluminescence signals (i). Box plots showing bioluminescent signals of leg bones from CTT NGL mice treated with exosomes from patients with ITP (n = 3) and healthy donors (n = 3) (ii). Quantification of selected gene mRNA expression in BM cells from the control (n = 6), HD‐exo‐treated (n = 8), and ITP‐exo‐treated groups (n = 8) (iii). *P* value significance represented by *, <0.05; **, <0.01; ***, <0.001; ****, <0.0001. F). Experimental methodology for examining the variances in exosome interactions between ITP patients (n = 3) and healthy donors (n = 3) by investigating changes in Treg cells (i). Quantification of CD4^+^FoxP3^+^ Treg‐like cells among CD45^+^CD3^+^ T cells (ii). *P* value significance represented by *, <0.05; **, <0.01; ***, <0.001; ****, <0.0001. G). A schematic illustration of the mechanism by which ITP‐specific exosomes influence platelet destruction.

Extracellular vesicles (EVs) have potential applications as diagnostic, prognostic, and therapeutic tools and are involved in immune regulation.^[^ ^8^ ^]^ To investigate the role of exosomes in ITP etiology, we incubated BM mononuclear cells (BMMCs) derived from healthy donors (HDs) with EXOs obtained from PB and BM plasma samples from both HDs and patients with ITP (**Figure** **1**A(i)). As anticipated, BMMCs showed greater uptake of exosomes from ITP patients than from HDs (Figure 1A(ii)). In addition, HD PBMCs were cultured with labeled platelets and exosomes to compare the BM exosomes from patients with ITP and healthy donors (Figure 1B(i)). The results revealed a higher incidence of 1,1′‐Dioctadecyl‐3,3,3′,3′‐tetramethylindocarbocyanine perchlorate [DIL]‐labeled monocytes in the ITP exosome group than in the HD exosome group (Figure 1B(ii)), suggesting that exosomes from patients with ITP facilitate platelet uptake (Figure 1B(iii); Figure S1A, Supporting Information). BM‐derived EXOs can be taken up by the liver, spleen, and BM, which are the sites of platelet destruction (Figure S1B, Supporting Information). ITP EXOs can increase the NF‐κB activation in some tissues by evaluating the NF‐κB‐response‐driven GFP expression, such as thymus, in vivo compared with HD EXOs (Figure S1C, Supporting Information). To evaluate the effects of exosomes from patients with ITP and HDs on thrombosis, we established a mouse model of carrageenan‐induced tail thrombosis (CTT) and found that exosomes from ITP patients enhanced thrombosis (Figure 1C; Figure S1D, Supporting Information). Activation of NF‐κB is essential in the initiation of inflammation, so we next assessed the bioluminescence signals driven by NF‐κB activity in CTT NGL mice (Figure 1D(i)). We observed an increase of NF‐κB activity in ITP‐exosome treated CTT NGL mice compared with HD‐exosome treated CTT NGL mice (Figure 1D(ii)). We next explored the NF‐κB activity in BM (Figure 1E(i)) and found that exosomes from patients with ITP can robustly promote inflammation (Figure 1E(ii)). In addition, several inflammation‐associated genes, including *Retn*, *Mif*, *Ptgs1*, *Ptgs2*, *Hif1a*, and *PF4*, were upregulated in BM cells from ITP‐exo‐treated mice compared with those from HD‐exo‐treated mice (Figure 1E(iii); Figure S1E, Supporting Information), indicating that ITP‐specific exosomes can be transferred into the BM microenvironment and further impair normal hematopoiesis. Our results highlighted that those exosomes from patients with ITP are the proinflammatory mediators.

Exosomes from patients with ITP decreased the proportions of regulatory T (Treg) cells in mouse spleens compared with those from HDs (Figure 1F; Figure S1F, Supporting Information), indicating that ITP exosomes have proinflammatory functions. These findings indicate that exosomes from patients with ITP, which enhance phagocytosis and induce inflammation may serve as potential targets for the treatment of ITP (Figure 1G).

### 1.2 Exosomes from Patients with ITP Exhibit Elevated Levels of Lectin Pathway Components of the Complement System

**Figure 2:**
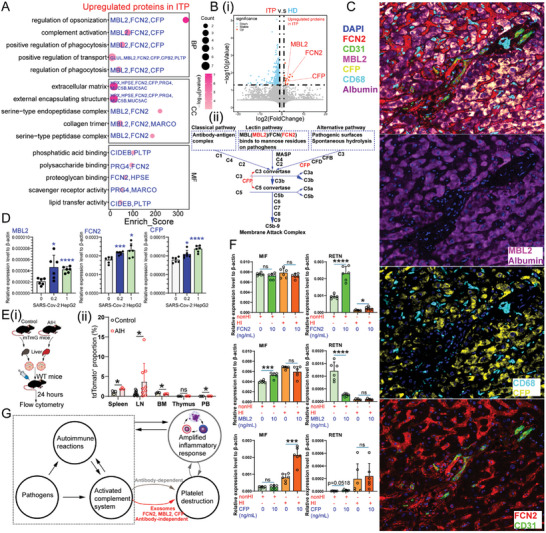
Exosome‐mediated lectin pathway of complement system enhances platelet destruction and immune responses through the transportation of MBL2, FCN2, and CFP proteins. A). Proteomic analysis of the BM plasma and plasma‐derived exosomes from individuals with primary immune thrombocytopenia. GO enrichment analysis was performed on the upregulated exosomal proteins in BM plasma samples from patients with ITP. BP, biological process; CC, cellular component; MF, molecular function. B). Volcano plots were used to display the selected upregulated proteins in exosomes from the BM plasma samples of patients with ITP compared to controls (i). A schematic overview of the complement pathways is presented, highlighting the master regulators of the complement system (ii). The differentially upregulated proteins, FCN2, MBL2, and CFP, are shown in red. The complement system serves as a pattern recognition and effector system for innate immunity and is capable of activation through the classical pathway by recognizing antibody clusters, the lectin pathway by sensing carbohydrate signatures, and the alternative pathway via the tick‐over mechanism. C). Immunofluorescence staining was performed on tumor‐adjacent liver tissue from patients diagnosed with human hepatocellular carcinoma (HCC). Hepatocyte marker (albumin), endothelial marker (CD31), Kupffer/macrophage marker (CD68), and selected proteins (CFP, MBL2, and FCN2) were assayed using immunofluorescence staining. D). Quantification of *FCN2*, *MBL2*, and *CFP* mRNA expression in the control and pseudovirus‐treated groups. *P* value significance represented by *, <0.05; **, <0.01; ***, <0.001; ****, <0.0001. E). Experimental methodology for examining the distribution of liver‐derived exosomes. Summary graphs showing the proportion of tdTomato^+^ cells in different tissues (thymus [normal, n = 3; AIH, n = 3], BM [normal, n = 3; AIH, n = 3], spleen [normal, n = 3; AIH, n = 3], PB [normal, n = 3; AIH, n = 3], and lymph nodes [LN] [normal, n = 8; AIH, n = 8]) from WT mice treated with normal liver‐derived exosome and AIH‐exosome treatments. *P* value significance represented by *, <0.05; **, <0.01; ***, <0.001; ****, <0.0001. F). *RETN* and *MIF* transcript levels were estimated after MBL2, FCN2, and CFP stimulation in BMMCs from HDs cultured with heat‐inactivated or normal plasma. G). Model of complement‐mediated platelet destruction in patients with ITP. Pathogens can trigger the complement activation of lectin pathways. Autoantibodies drive classical complement activation and mediate platelet destruction. In addition to classical complement activation, our findings revealed that the lectin pathway is a potential driver of platelet destruction mediated by exosomal FCN2, MBL2, and CFP. These pathways may further enhance the inflammatory responses. Complement activation is directly involved in platelet destruction and cross‐talk with amplified inflammatory responses including acquired immunity.

To elucidate the distinctions between ITP exosomes and those from HDs, label‐free quantitative proteomic analysis was conducted on exosomes isolated from BM plasma samples of both HDs and ITP patients (Figure S2A, Supporting Information). Gene ontology (GO) analysis revealed an increase in immune‐related proteins within the ITP EXOs, suggesting their involvement in phagocytosis and complement activation (**Figure** **2**A; Figure S2B, Supporting Information). Upregulation of the complement‐related proteins MBL2, FCN2, and CFP was observed in ITP EXOs, suggesting a close association between microorganisms and ITP (Figure 2B(i)). MBL2 can spike the spike protein of SARS‐CoV‐2 and activate the lectin pathway of complement activation,^[^ ^9^ ^]^ a process linked to COVID‐19‐associated thrombosis.^[^ ^10^ ^]^ FCN2, identified as a pathogenic factor, is implicated in thrombocytopenia observed in patients with SLE.^[^ ^11^ ^]^ CFP, also known as properdin, functions as a positive regulator of the complement cascade by stabilizing C3 and C5 convertases. Anti‐platelet antibodies have been shown to trigger the classical complement pathway, which is a key pathophysiological mechanism in ITP.^[^ ^12^ ^]^ Our findings suggest that EVs may also play a role in ITP pathophysiology through complement pathways independent of antibodies (Figure 2B(ii)). Interestingly, our analysis of plasma samples did not reveal elevated levels of FCN2, MBL2, and CFP compared with HDs, as determined by label‐free quantitative proteomic analysis (Figure S2A,C, Supporting Information). Some samples from patients with ITP exhibit elevated concentrations of immunoglobulins, indicating the presence of activated B/plasma cells (Figure S2D, Supporting Information).

To identify the potential sources of FCN2‐, MBL2‐, and CFP‐carrying EXOs, we analyzed the expression levels of these genes using the Tabula Sapiens database (https://tabula‐sapiens‐portal.ds.czbiohub.org/). Interestingly, high levels of *FCN2* were found in endothelial cells of the hepatic sinusoid, whereas hepatocytes showed high expression of *MBL2* and myeloid lineages, including monocytes, neutrophils, and macrophages, exhibit high expression of *CFP* (Figure S3A, Supporting Information). Macrophages derived from the spleen (SP) and liver exhibited elevated expression levels of *CFP* (Figure S3B, Supporting Information). Additionally, we investigated the expression profiles of *Fcna*, *Cfp*, and *Mbl2* in the mouse liver, revealing that *Fcna* originates primarily from stellate cells, *Cfp* from Kupffer cells, and *Mbl2* from hepatocytes (Figure S3C, Supporting Information). The BM, SP, and liver are critical sites of platelet destruction, indicating that the lectin pathway of the complement system is an important regulator of platelet homeostasis. In this study, we analyzed the protein expression profiles of MBL2, FCN2, and CFP in human liver tissues using immunofluorescence techniques (Figure 2C). Our results showed that the MBL2 protein was predominantly expressed in albumin^+^ hepatocytes, consistent with its mRNA expression pattern. FCN2 protein was observed in in endothelial cells as well as in hepatic stellate cells lacking albumin expression. Interestingly, the CFP protein was expressed in hepatocytes rather than in CD68^+^ immune cells (Figure S3D, Supporting Information).

Pathogen infections, such as SARS‐CoV‐2,^[^ ^13^ ^]^ bunyavirus,^[^ ^14^ ^]^ and *Helicobacter pylori*,^[^ ^15^ ^]^ have been shown to activate platelets and induce inflammation. In this study, we investigated the relationship between pathogens and lectin pathways. We found that stimulation with the SARS‐CoV‐2 pseudovirus led to increased expression of *MBL2* and *FCN2* in the leukemia monocytic cell line THP‐1 and THP‐1‐derived macrophage‐like cells (Figure S4A, Supporting Information). Additionally, stimulation with the SARS‐CoV‐2 pseudovirus resulted in increased expression of *MBL2*, *FCN2*, and *CFP* in the hepatoblastoma‐derived cell line Hep‐G2 (Figure 2D). However, this specific transcriptional pattern has not been observed in human PB mononuclear cells (PBMCs). Subsequently, we investigated the alterations in *FCN2*, *MBL2*, and *CFP* transcripts following pseudovirus stimulation in various macrophage types, including pro‐inflammatory (M1), non‐activated (M0), and anti‐inflammatory (M2) subsets (Figure S4B, Supporting Information). Pseudovirus stimulation modestly enhanced the mRNA expression levels of *MBL2*, *FCN2*, and *CFP* in PBMC‐derived M0 and M2 subtypes under specific conditions, whereas no such effect was observed in the M1 subtype. Next, we used a mouse model of autoimmune hepatitis (AIH) to examine the crosstalk between liver‐derived exosomes and the immune/hematopoietic system (Figure 2E(i)). The upregulated proportions of tdTomato^+^ cells in the LN, spleen, and PB samples from AIH EXO‐treated groups indicated that abnormal EXOs could be delivered to other organs and involved in immune regulation (Figure 2E(ii)). Interestingly, the expression levels of *Cfp*, *Mbl2*, *Fcna*, and *Fcnb* mRNA were not upregulated in the livers of AIH mice compared with their counterparts (Figure S4C, Supporting Information). We treated BMMCs with FCN2, MBL2, and CFP and found that FCN2 enforced the upregulation of *RETN* transcripts (Figure 2F). Furthermore, CFP treatment could drive the upregulation of *MIF* mRNA under the condition of heat‐inactivation of plasma and MBL2 treatment also increased the expression level of MIF under the condition of non‐heat‐inactivation of plasma (Figure S4D, Supporting Information). In summary, abnormal liver immune responses appear to be associated with ITP through the exosome‐mediated activation of the lectin pathway of the complement system (Figure 2G).

### 1.3 Single‐Cell Transcriptomics Uncovers Novel Pathways of Complement‐Mediated Immunoregulation in Platelet Destruction

**Figure 3:**
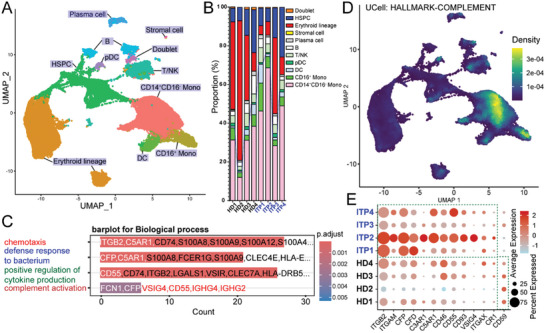
Landscape of BM mononuclear cells in patients with ITP. A). Uniform manifold approximation projection (UMAP) visualization of single‐cell transcriptional states obtained from BM mononuclear cells of patients with ITP compared with HDs. Distinct colors represent individual cell types determined based on gene expression: HSPC, hematopoietic stem and progenitor cells; T/NK, T and NK cells; pDC, plasmacytoid dendritic cells; DC, dendritic cells; Mono, monocytes. B). Analysis of proportions of different cell types in each sample. C). Selected lists of DE genes for ITP patient‐derived BMMC compared to HD‐derived BMMC were analyzed by GO analysis and annotated using GO terms. Graphs depicting selected GO terms, including complement‐associated genes. D). UMAP of BMMC showing the scores of “UCell: HALLMARK‐COMPLEMENT.” E). Bubble plot of complement pathway associated gene expression in myeloid population, including CD14^+^CD16^−^ monocytes, CD16^+^ monocytes, and DC from patients with ITP versus healthy donors.

Increased levels of complement elements indicate heightened innate immune responses, prompting an investigation of the transcriptional profile of immune cells at the single‐cell level. scRNA‐seq was conducted on BMMCs from four ITP patients and four HDs. Cell clusters were manually classified into ten distinct cell types based on their unique gene expression profiles (**Figure** **3**A; Figure S5A, Supporting Information). The proportion of monocytes, specifically CD14^+^CD16^−^ and CD16^+^ monocytes, was significantly higher in patients with ITP compared with HDs (Figure 3B). Furthermore, an activated immune state of B/plasma and T cells was identified (Figure S5B,C, Supporting Information), indicating the involvement of adaptive immunity in ITP. Differential gene expression analysis was conducted between BMMCs from ITP patients and HDs, followed by GO analysis. Numerous complement‐associated genes, such as *ITGB2*, *C5AR1*, *CFP*, *CD55*, *FCN1*, and *VSIG4*, are involved in various biological processes, including “complement activation” and “defense response” (Figure 3C). Several complement‐associated genes, such as *CFD* and *CFP*, are upregulated in BMMCs obtained from patients with ITP (Figure S5D, Supporting Information). Monocytes display elevated transcriptional levels of the complement system (Figure 3D; Figure S5E, Supporting Information). The mRNA transcripts of *ITGB2* (CD18), *ITGAM* (CD11b), *CFP*, *CFD*, and *C5AR1* were upregulated in monocytes derived from patients with ITP, suggesting the enhancement of complement‐mediated phagocytosis in these monocytes (Figure 3E). Hence, the scRNA‐seq atlas of BMMCs from ITP patients highlights the significance of complement‐associated transcripts in the pathogenesis of ITP, warranting further validation through robust evidence.

### 1.4 Downregulation of CD55 serves as a Mechanism for Complement‐Mediated Immune Activation and a Potential Therapeutic Target in ITP

**Figure 4:**
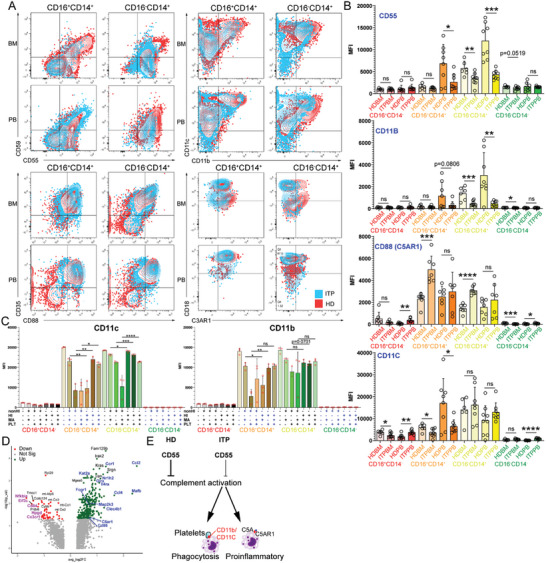
The ITP monocytes exhibit distinct characteristics of dysfunctional complement‐associated regulatory networks. A). Representative flow cytometry plots showing the fluorescence intensity of CD59, CD55, CD11c, CD11b, CD35, and CD88 in paired BMMC and PBMC samples from patients with ITP and HDs. B). Summary graphs showing themedian fluorescence intensity (MFI) of CD55, CD11b, and CD88 in different subsets of BMMC and PBMC from patients with ITP and HDs. P value significance represented by *, <0.05; **, <0.01; ***, <0.001; ****, <0.0001. C). Summary graphs showing the median fluorescence intensity (MFI) of CD11c and CD11b in different cell subsets of PBMC from healthy donors (n = 3) after co‐incubation with platelets and/or mannan. P value significance represented by *, <0.05; **, <0.01; ***, <0.001; ****, <0.0001. D). Volcano plot showing upregulated and downregulated genes in monocytes after C5a treatment in a mouse model. E). Weaker CD55 levels in patients with ITP indicate more sensitive complement activation. Activated complement components enhance platelet destruction by utilizing CD11b/CD11c mediated phagocytosis. Furthermore, innate immune cells can increase inflammatory responses via the C5A‐C5AR1 axis.

To strengthen our argument regarding the role of complements in ITP, we conducted FCM analysis to detect surface complement‐associated proteins (Figure S6A, Supporting Information). Although the levels of CD59 and CD46 surface proteins remained consistent, a significant decrease in CD55 surface protein levels was observed in BM‐ and PB‐derived monocytes from patients with ITP (**Figure** **4**A). FCM analysis indicated a decrease in CD11b and CD11c (ITGAX) surface proteins as well as a slight reduction in CD18 in monocytes from patients with ITP, which contradicts the upregulation of relevant transcripts identified by scRNA‐seq (Figure 4B). Changes in the cell surface levels of CR3 (CD11b/CD18) and CR4 (CD11c/CD18) may be attributed to endocytosis induced by stimulating agents.^[^ ^16^ ^]^ To ascertain the relationship between changes in CR3 and CR4 and platelet destruction, PBMCs from HDs were cultured with platelets incubated with mannan in the presence of human serum, with or without heat inactivation. The addition of platelets and human serum without heat inactivation resulted in reduced CD11b and CD11c surface protein levels in monocytes (Figure 4C). These findings indicate that the decrease in CD11b and CD11c surface protein levels may be linked to ongoing phagocytosis, potentially explaining the discrepancy between transcript and surface protein levels.

Monocytes derived from patients with ITP exhibit significant upregulation of C5AR1 and a slight increase in CR1, but not C3AR1 (Figure 4B). Activation of the C5a‐C5aR1 axis is a potent inducer of proinflammatory responses, suggesting that hyperactivation of the lectin pathway in the complement system may enhance immune activation through the production of C5a. Subsequent analysis of a publicly available dataset on cytokine‐driven cellular polarization revealed that treatment with C5a induced the upregulation of proinflammatory genes, such as *Ccl2*, *Ccl4*, *Fcgr1*, *Ccr1*, *C5ar1*, and *CD86*, while concurrently downregulating anti‐inflammatory genes including *Nfkbia*, *Eif3c*, *Cd84*, *Hpgd*, and *Cx3cr1* (Figure 4D). Therefore, we propose a model that links the lectin pathway to complement system dysfunction in patients with ITP based on the exosome‐mediated lectin pathway, decreased CD55 levels, and increased C5AR1 expression (Figure 4E; Figure S6B, Supporting Information).

### 1.5 Resistin as a Proinflammatory Cytokine Involved in the Pathological Processes of ITP

**Figure 5:**
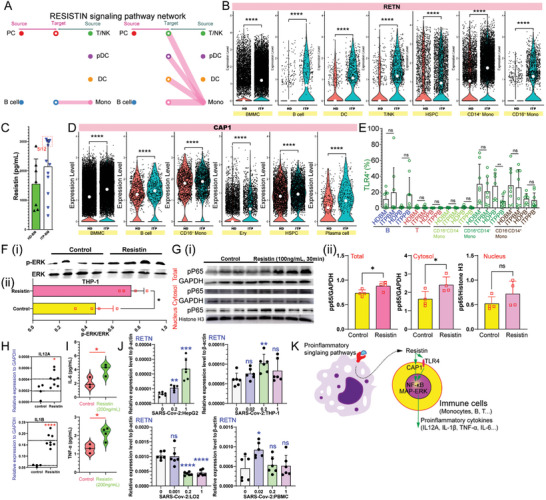
Upregulation of resistin occurs in the immune cells of ITP patients. A). CellChat analysis of the signaling milieu in the RESISTIN signaling pathway network. B). Violin plots illustrating the distribution of RETN expression across various subsets. *P* value significance represented by *, <0.05; **, <0.01; ***, <0.001; ****, <0.0001. C). Resistin protein levels in BM plasma (HD, n = 7; ITP, n = 12) were quantified by using ELISA. D). Violin plots illustrating the distribution of CAP1 expression across various subsets. *P* value significance represented by *, <0.05; **, <0.01; ***, <0.001; ****, <0.0001. E). Histograms showing the expression levels of cellular surface TLR4 in various immune cellsubsets. *P* value significance represented by *, <0.05; **, <0.01; ***, <0.001; ****, <0.0001. F). Protein levels of ERK and pERK in whole‐cell extracts of THP‐1 control (n = 4) and resistin‐treated THP‐1 (n = 4) groups (i). Histograms showing quantification of the p‐ERK/ERK ratio (ii). *P* value significance represented by *, <0.05; **, <0.01; ***, <0.001; ****, <0.0001. G). Protein levels of pP65 and GAPDH in the whole‐cell extract and cytoplasmic and nuclear fractions of THP‐1 control groups (n = 4) and resistin‐treated groups (n = 4) (i). Histograms showing quantification of the pP65/GAPDH ratio (ii). P value significance represented by *, <0.05; **, <0.01; ***, <0.001; ****, <0.0001. H). *IL12A*, *TNFSF10*, and *IL1B* transcript levels in THP‐1 cells after resistin stimulation (control, n = 4; treated group, n = 8). *P* value significance represented by *, <0.05; **, <0.01; ***, <0.001; ****, <0.0001. I). Levels of IL‐6 (control, n = 3; treated group, n = 3) and TNF‐α (control, n = 3; treated group, n = 4) in supernatants of THP‐1 cultures, measured using ELISA after treatment with resistin. J). Transcript levels of RETN in various cell types treated with pseudovirus. Six independent experiments were performed for the statistical analyses. *p < 0.05, **p < 0.01, ***p < 0.001, ****p < 0.0001. K). Inflammatory processes are potential drivers of resistin expression, which can enhance inflammatory signaling via NF‐κB and ERK signaling pathways, resulting in inflammatory cytokine production.

To further understand the interactions between the different hematopoietic subpopulations, we conducted a cell communication analysis using CellChat. Our findings indicated that monocytes, particularly CD14^+^ monocytes, are the primary source of resistin (RETN) (**Figure** **5**A; Figure S7A, Supporting Information). Resistin upregulates P‐selectin expression in platelet via the p38 MAPK pathway, leading to enhanced platelet activation.^[^ ^17^ ^]^ Ligands from monocytes can activate the resistin signaling pathway in nearly all immune subsets. Notably, higher levels of *RETN* mRNA were detected not only in monocytes, but also in various other major cell subsets such as B cells, DC, T/NK, HSPC, and other cell subsets in patients with ITP than in HDs (Figure 5B; Figure S7B, Supporting Information). Interestingly, *RETN* transcripts appeared to be decreased in BM granulocytes from patients with ITP compared with those from HDs (Figure S7C, Supporting Information). The expression levels of *RETN* were evaluated in diverse tissues and organs and revealed high levels of expression in the BM (Figure S7D, Supporting Information). Additionally, elevated concentrations of RETN were observed in BM plasma samples from patients with ITP compared with PB samples (Figure 5C). Previous studies have indicated that CAP1, the receptor of resistin for resistin, is highly expressed in platelets.^[^ ^18^ ^]^ Our findings demonstrate that *CAP1* mRNA was predominantly expressed at high levels in most subsets, with lower expression in erythroid lineages (Figure S7E, Supporting Information). Our study revealed that the expression of *CAP1* mRNA was increased in various cell subsets, including B cells, CD16^+^ monocytes, erythroid lineages, plasma cells, HSPCs, and other cell subsets, in patients with ITP (Figure 5D; Figure S7F, Supporting Information). Additionally, TLR4, a potential receptor for resistin, was predominantly expressed in monocytes, T/NK cells, and other immune cell types (Figure S8A, Supporting Information). Interestingly, the surface TLR4 protein in CD14^+^CD16^+^ monocytes from patients with ITP was downregulated (Figure 5E; Figure S8B, Supporting Information), suggesting the possibility of receptor‐mediated endocytosis upon ligand binding.^[^ ^19^ ^]^

Resistin has been shown to enhance the inflammatory response of monocytes and induce low‐grade inflammation through the activation of the Erk and cAMP‐PKA‐NF‐κB pathways.^[^ ^20^ ^]^ Resistin treatment increased the levels of phosphorylated ERK (Figure 5F). Furthermore, resistin stimulation also enhanced phosphorylated P65 (Figure 5G; Figure S8C, Supporting Information). The Erk and NF‐κB signaling pathways play a critical role in promoting proinflammatory responses in immune cells. Following resistin stimulation, there was an upregulation of mRNA expression levels of *IL12A* and *IL1B* in the THP‐1 cell line, while *TNFSF10* expression was downregulated (Figure 5H; Figure S8D, Supporting Information). Treatment with resistin significantly increased the secretion of IL‐6 and TNF‐α proteins in the supernatant of THP‐1 cells (Figure 5I; Figure S8E, Supporting Information). Our findings were further confirmed in various cell lines using qPCR, including the pre‐B ALL cell line NALM6, human acute myeloid leukemia cell line HL60, human T leukemic cell line Jurkat, and multiple myeloma cell line RPMI‐8826 (Figure S8F, Supporting Information).

Subsequently, we investigated the regulatory factors influencing resistin production. The SARS‐CoV‐2 pseudovirus was utilized to simulate pathogens, revealing an enhancement in *RETN* expression in HepG2 cells, THP‐1 cells, and PBMCs (Figure 5J; Figure S9A, Supporting Information). Subsequent analysis of public datasets pertaining to cytokine‐driven cellular polarization of cell types indicated that neutrophils could upregulate mouse *Retn* mRNA in response to various cytokines in vivo, including 4‐1BBL, BAFF, cardiotrophin‐1, CD27L, CD40L, G‐CSF, GITRL, IFNI2, IFNk, IL‐10, IL‐12, IL17D, IL17E, IL17F, IL20, IL21, IL23, IL24, IL30, IL34, IL36RA, IL5, IL7, IL9, Leptin, Noggin, OX40L, Persephin, Prolactin, RANKL, SCF, TGFB1, TL1A, and TSLP. LIF and IL‐2 upregulated mouse *Retn* expression in monocytes (Figure S9B, Supporting Information). Additionally, lipopolysaccharide (LPS), a known pathogen‐associated molecular patterns (PAMPs), were observed to increase *RETN* mRNA expression (Figure S9C, Supporting Information). These findings indicate that increased expression of RETN may be a consequence of various proinflammatory signaling pathways. Considering the potential origin of resistin, our results suggest that it serves as a marker of inflammatory response in patients with ITP (Figure 5K).

### 1.6 Resistin Plays a Role in Combating GC‐Induced Immunosuppression and is Involved in AA Metabolism

**Figure 6:**
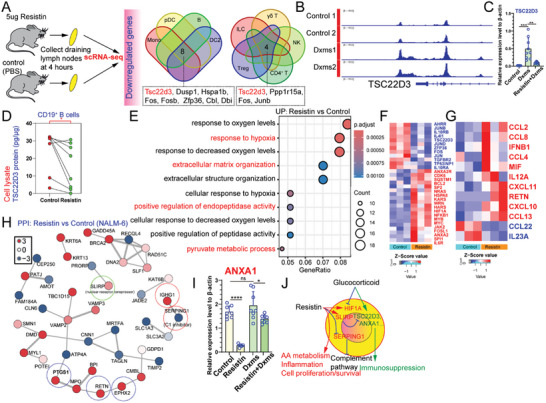
Identification of resistin‐programmed gene expression programs that characterize downregulated TSC22D3 involving in resistance of Dex treatment. A). Venn plots of downregulated differentially expressed genes (DEGs) in various lymph node cell types from resistin‐treated mice compared with control mice injected with PBS. pDC, plasmacytoid dendritic cell; cDC2, type 2 conventional dendritic cell; mono, monocytes; B, B cells; ILC, innate lymphoid cells; Treg, regulatory T cells; NK, natural killer cells; γδ T, TCRγδ T cells. B). Genome browser tracks of CUT& Tag signals at the representative target gene loci. Acetyl‐histone H3 (Lys27) (D5E4) XP Rabbit mAb was used to recognize the endogenous levels of histone H3 only when acetylated at Lys27. C). TSC22D3 transcript level estimated after dxms combined with or without resistin stimulation in THP‐1 cells. In all panels, n = 9 for each group. *P* value significance represented by *, <0.05; **, <0.01; ***, <0.001; ****, <0.0001. D). CD19^+^ B cells were isolated from HD PBMC (n = 8) and incubated with resistin, and TSC22D3 protein levels in total proteins from cell lysates were measured using ELISA. *P* values determined by two‐tailed ratio paired T test, represented by *, <0.05; **, <0.01; ***, <0.001; ****, <0.0001. E). GO enrichment analysis of genes upregulated in resistin‐treated THP‐1 cells using bulk RNA‐Seq. F). Heatmap showing the relative expression levels of selected genes in THP‐1 cells treated with MIF (n = 3) or without resistin (n = 3). G). Heatmap showing relative expression levels of selected chemokine and cytokine‐associated genes in THP‐1 cells treated with MIF (n = 3) or without resistin (n = 3). H). Protein–protein interactions (PPI) networks of differentially expressed proteins in NALM6 cells following resistin treatment. I). *ANXA1* transcript level estimated after resistin and/or dxms stimulation of THP‐1 cells. *P* value significance represented by *, <0.05; **, <0.01; ***, <0.001; ****, <0.0001. J). A schematic representation of resistin and dxms participation in immune regulation.

To identify genes that specifically regulate resistin‐induced immune regulation, we analyzed scRNA‐seq datasets from the Immune Dictionary. This resource contains a comprehensive collection of single‐cell transcriptomic profiles of over 17 immune cell types in response to 86 cytokines, including resistin. Surprisingly, our findings revealed that resistin treatment led to a decrease in the expression of *Tsc22d3* across various cell types, including B cells, monocytes, DC2, pDC, ILC, γδ T, NK, Tregs, and CD4^+^ T cells (**Figure** **6**A). TSC22D3, acting as a mediator in the therapeutic mechanism of GC, can be stimulated by both GC and IL‐10, leading to anti‐inflammatory and immunosuppressive effects by inhibiting pro‐inflammatory molecules such as NF‐κB.^[^ ^21^ ^]^ We performed cut&tag analysis and identified enhanced transcriptional activation of TSC22D3 triggered by dexamethasone (dxms) (Figure 6B). The upregulation of TSC22D3 in PMA‐stimulated THP‐1 cells by dxms was inhibited by resistin (Figure 6C). Rilzabrutinib, a Bruton's tyrosine kinase inhibitor, has been identified as an effective and well‐tolerated treatment for ITP, suggesting that B cells are involved in ITP pathogenesis. GC and TSC22D3 induce apoptosis in B cells.^[^ ^22^ ^]^ Alterations in the TSC22D3 protein levels in human CD19^+^ B cells following resistin stimulation were also investigated. Consistent with the changes in *Tsc22d3* mRNA levels, resistin treatment decreased TSC22D3 protein expression (Figure 6D). We also performed bulk RNA‐seq analysis of THP‐1 cells treated with resistin and found that resistin treatment altered immune‐related gene expression patterns (Figure S10A, Supporting Information). Gene Ontology (GO) analysis of upregulated genes in resistin‐treated groups revealed that the resistin can robustly affect the several important biological processes, including “response to hypoxia,” “extracellular matrix organization,” “positive regulation of endopeptidase activity,” and “pyruvate metabolic process” (Figure 6E). Consistently, we found that resistin treatment downregulated *TSC22D3* transcripts in THP‐1 cells and upregulated proliferation‐ and survival‐associated genes, including *SQSTM1*, *BCL2*, *NRAS*, *KRAS*, *HRAS*, *HSPA8*, *HIF1A*, *MYB*, and *MYC* (Figure 6F). Resistin treatment upregulated proinflammatory cytokines (*CCL2*, *CCL8*, *IFNB1*, *CCL4*, *MIF*, *IL12A*, *CXCL11*, *RETN*, *CXCL10*, and *CCL13*) and downregulated anti‐inflammatory cytokines (*CCL22* and *IL23A*) (Figure 6G). Mass spectrometry‐based proteomics was used to evaluate the resistin‐induced responses. Resistin downregulated the protein levels of SERPING1 and upregulated those of several key proteins, including IGHG1 (IgG1 heavy chain), EPHX2, RETN, PTGS1, and SLIRP (Figure 6H). Interestingly, the majority of autoantibodies found in patients with ITP belong to the IgH1 subclass, leading to the activation of the classical complement pathway.^[^ ^23^ ^]^ Additionally, Fc galactosylation of anti‐platelet human IgG1 alloantibodies has been shown to interact with C1q, resulting in activation of the classical complement pathway,^[^ ^24^ ^]^ a process that can be inhibited by SERPING1. Furthermore, the enzymes PTGS1 and EPHX2 are involved in AA metabolism, contributing to platelet destruction. Notably, SLIRP has been identified as a corepressor of nuclear receptors and potentially plays a role in GC resistance.^[^ ^25^ ^]^ To enhance the role of resistin in mediating resistance to dxm treatment, various genes associated with the GC response were investigated (Figure S10B, Supporting Information). Resistin markedly decreased the expression of the anti‐inflammatory biosynthetic mediator ANXA1 in the presence of dxms (Figure 6I). These findings indicate that resistin antagonizes the immunoregulatory effects of endogenous and exogenous GC. Furthermore, treatment with dxms upregulated *ALOX5* transcription, which was attenuated by resistin. ALOX5 plays a role in AA metabolism, lipid peroxidation, and cell death, potentially by regulating hyperactivated immune cells.^[^ ^26^ ^]^ Taken together, the upregulated resistin is implicated in antibody‐mediated complement activation, reprogramming of AA metabolism, and resistance to dxms treatment (Figure 6J).

### 1.7 MIF is Linked to ITP and Resistin‐Mediated Immune Responses

**Figure 7:**
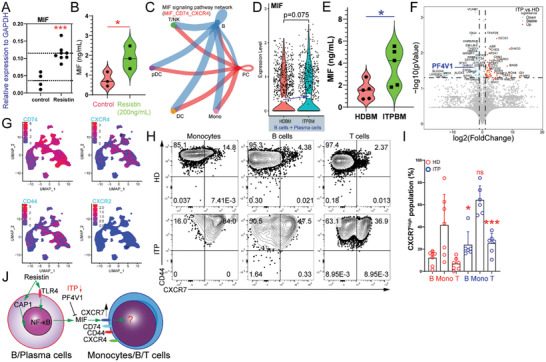
MIF contributes to ITP pathogenesis by exerting inflammatory effects on immune cells. A). MIF transcript levels were estimated after resistin stimulation in THP‐1 cells. *P* value significance represented by *, <0.05; **, <0.01; ***, <0.001; ****, <0.0001. B). MIF protein levels were estimated after resistin stimulation in supernatants of THP‐1 cultures. *P* value significance represented by *, <0.05; **, <0.01; ***, <0.001; ****, <0.0001. C). CellChat analysis of the signaling milieu in the MIF signaling pathway network. D). Violin plots illustrating the expression distribution of MIF in B cells and plasma cells across various groups. E). MIF protein levels in the BM plasma were quantified using ELISA. *P* value significance represented by *, <0.05; **, <0.01; ***, <0.001; ****, <0.0001. F). Volcano plot (ITPBM versus HDBM) of differentially expressed proteins. Differences in the abundance of proteins between ITP patients’ BM plasma samples and healthy donor BM plasma samples were assessed by proteomics profiling. The MIF‐associated protein PF4V1 is highlighted in blue. G). Feature plots of the potential MIF receptors (*CD74*, *CXCR4*, *CD44*, and *CXCR2*) in UMAP. H). Representative flow cytometry plots showing the fluorescence intensities of CD44 and CXCR7 in BMMC samples from patients with ITP and healthy donors. I). Proportion of CXCR7^high^ subsets in BM B cells, T cells, and monocytes from patients with ITP (n = 6) versus healthy donors (n = 7). *P* value significance represented by *, <0.05; **, <0.01; ***, <0.001; ****, <0.0001. J). A schematic representation of the participation of the MIF signaling pathway in immune regulation.

As **Figure** **7**A shows, resistin could increase the expression levels of *MIF* (Figure S8F, Supporting Information), confirmed at the protein level using enzyme‐linked immunosorbent assay (ELISA) (Figure 7B). CellChat analysis revealed that B‐ and plasma cell‐derived MIF can facilitate the signaling of various immune cell types (Figure 7C). Notably, B cells and plasma cells obtained from patients with ITP exhibited elevated levels of *MIF* transcripts compared with those from HDs (Figure 7D). Furthermore, primary CD19^+^ B cells exhibited increased MIF protein expression following resistin treatment (Figure S11A, Supporting Information). Measuring BM plasma MIF concentrations in samples from five HDs and five patients with ITP using ELISA, we found significantly higher levels of MIF in patients with ITP than in HDs (Figure 7E). *MIF* mRNA was observed in both HSPC and early erythroid progenitors (Figure S11B, Supporting Information), and ITP patient‐derived HSPCs exhibited higher expression level of *MIF* than HDs (Figure S11C, Supporting Information). Furthermore, plasma proteomic analysis has revealed a decrease in PF4V1, a suppressive molecule of MIF, in the BM plasma of patients with ITP (Figure 7F).

Subsequent investigation of the expression profiles of MIF receptors (CD74, CD44, CXCR4, and CXCR2) indicated that *CXCR4*, *CD74*, and *CD44* mRNA were detectable in all cell subsets (Figure 7G). We examined the expression levels of CD74, CD44, CXCR4, and CXCR7 surface proteins (Figure S11D, Supporting Information). B cells displayed higher levels of CD74 surface protein than T cells and monocytes (Figure S11E, Supporting Information). CXCR4 surface protein was present at elevated levels in BM B cells, monocytes, and T cells. Notably, the proportions of CXCR7^high^ subsets were significantly increased in monocytes, B cells, and T cells of patients with ITP compared with those in HDs (Figure 7H,I). BM plasma CXCL12 concentrations in patients with ITP were not significantly different from those in HDs (Figure S11F, Supporting Information), suggesting that MIF may play a dominant role in the inflammatory response mediated by CXCR2/CXCR4/CD44/CD74/CXCR7. Furthermore, in addition to activating Erk1/2 signaling through CD74 and CXCR4, MIF can induce the downstream activation of PI3K‐Akt and phosphorylation‐mediated inactivation of the proapoptotic protein BAD through CXCR7.^[^ ^27^ ^]^ These findings indicate that MIF contributes to platelet destruction and dysfunctional immune regulation in patients with ITP (Figure 7J).

### 1.8 The Downregulation of TP53 Expression by MIF Contributes to the Survival of Activated Immune Cells

**Figure 8:**
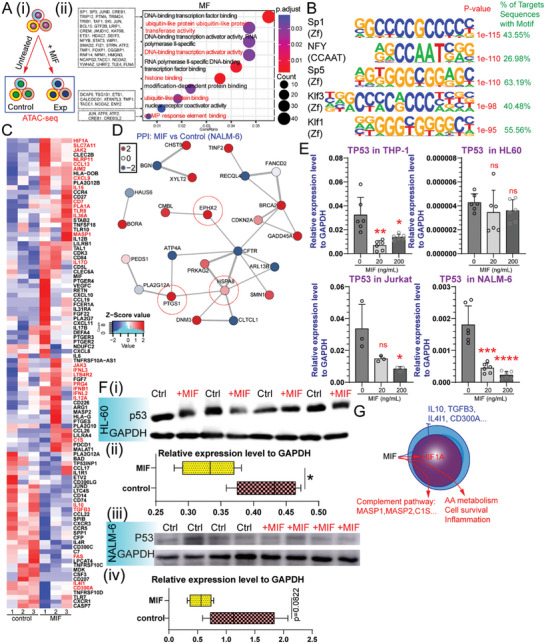
MIF alters the immune regulation and immune cell fates. A). Schematic representation of the experimental approach used for ATAC‐seq of HD PBMC after MIF stimulation. B). MIF‐treatment enriched motifs containing *Sp1*, *NFY*, *Sp5, Klf3*, and *Klf1* factors. C). Heatmap showing the relative expression levels of selected genes in THP‐1 cells treated with (n = 3) and without MIF (n = 3). D). Protein–protein interactions (PPI) networks of differentially expressed proteins in NALM6 cells after MIF treatment. E). qPCR validation of relative gene expression of *TP53* in various cell lines (THP‐1, n = 6; HL60, n = 6; Jurkat, n = 3; NALM6, n = 6) after MIF stimulation. *P* value significance represented by *, <0.05; **, <0.01; ***, <0.001; ****, <0.0001. F). Western blot analysis of TP53 protein levels in HL‐60 cells after stimulation with vehicle or MIF (i). Histograms showing the quantification of TP53 protein (n = 3 for each group) (ii). G). Schematic representation of the mechanism by which MIF exacerbates autoimmune responses.

To investigate the potential inflammatory effects of MIF on immune regulation, various cell lines (THP‐1, HL60, Jurkat, and NALM6) were exposed to MIF. MIF treatment resulted in distinct gene expression profiles in the different cell lines (Figure S12A, Supporting Information). To elucidate the mechanisms by which MIF mediates gene regulation, assay for transposase‐accessible chromatin sequencing (ATAC‐seq) was performed on MIF‐treated PBMCs (**Figure** **8**A(i)). Next, we conducted GO analysis of the genes linked to differential peaks in PBMCs treated with MIF compared to untreated PBMCs. GO term analysis identified molecular function (MF) including DNA‐binding transcription factor binding, nuclear receptor coactivator activity, and cAMP response element binding, biological processes (BP) such as histone modification, and cellular components (CC) such as the mitochondrial matrix, all of which are implicated in MIF‐mediated immune cell fates (Figure 8A(ii); Figure S12B, Supporting Information). The regulatory patterns of several key genes including *SP1*, *SP3*, *JUND*, *CREB1*, *TRIP12*, *PTMA*, *TRIM24*, *TRIB1*, *TAF1*, *SKI*, and *JUN* were altered. Interestingly, these genes were associated with p53‐mediated biological processes.^[^ ^28^ ^]^ Motif enrichment analysis indicated that MIF‐induced transcriptional activity relies on regulatory networks mediated by *Sp1*, *NFY*, *Sp5*, *Klf3*, *Klf1*, *Sp2*, *KLF5*, *Klf9*, *Klf6*, and *KLF14* (Figure 8B; Figure S12C, Supporting Information). MIF treatment increases the expression of inflammation‐associated genes, including *C1S*, *JAK2*, *NLRP11*, *CCL13*, *TLR8*, *MASP1*, and *IL17D*, and decreases that of anti‐inflammatory genes, including *IL10*, *TGFB3*, and *IL4I1* (Figure 8C). Proteomic analysis revealed that treatment with MIF had a significant effect on cell cycle, survival, proliferation (TINF2, RECQL4, BRCA2, CDKN2A, and GADD45A), and cell death (HSPA8) (Figure 8D). These findings are closely associated with the biological processes regulated by P53.^[^ ^29^ ^]^ Furthermore, the activation of SP1, NFY, KLF5, KLF9, and KLF6 strongly correlated with the functional role of TP53.^[^ ^30^ ^]^ Subsequent analysis of gene expression levels demonstrated a decrease in *TP53* mRNA expression following MIF treatment (Figure 8E), which was consistent with the observed anti‐apoptotic effects mediated by the MIF‐CXCR7 axis (Figure 7J). Western blot analysis revealed that treatment with MIF (24 h, 20 ng mL^−1^) resulted in the downregulation of P53 protein in NALM6 and HL60 cells (Figure 8F; Figure S12D, Supporting Information). These findings were consistent with a previous study.^[^ ^31^ ^]^ Although MIF does not promote cell proliferation in vitro (Figure S12E, Supporting Information), it plays a role in apoptosis (Figure S12F, Supporting Information). These results demonstrate that MIF enhances both autoimmune response and immune cell survival (Figure 8G).

### 1.9 MIF Enhances the AA Metabolism and Counteract the Effects of GC Therapy

**Figure 9:**
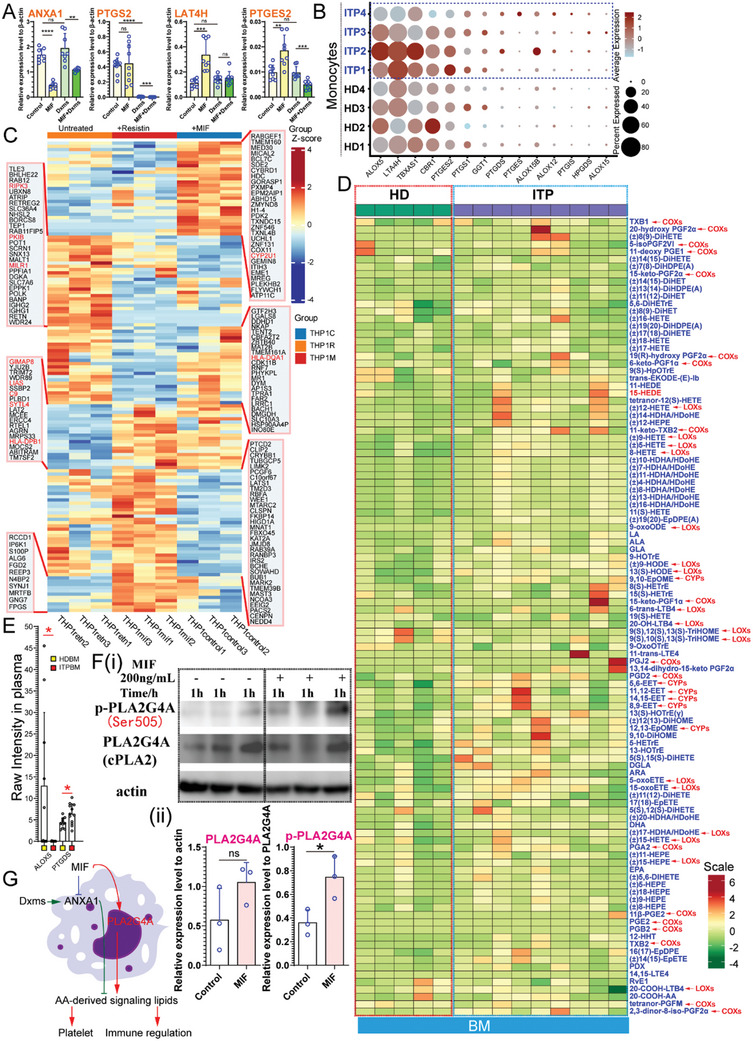
MIF changes arachidomic acid metabolism and causes dysfunction of platelet and immune regulation. A). qPCR analysis of selected genes in THP‐1 cells after stimulation with vehicle, dxms, MIF, and the combination of MIF and dxms (*ANXA1*, n = 8 per group; *PTGS2*, n = 9 per group; *LAT4H*, n = 8 per group; *PTGES2*, n = 8 per group). B). Bubble plot of arachidomic acid metabolism associated gene expression in monocytes from patients with ITP versus healthy donors. C). Heatmap of differentially expressed proteins. Differences of protein abundance between resistin‐treated THP‐1 cells, MIF‐treated THP‐1 cells, and untreated controls were assessed by proteomic profiling. D). Arachidomic acid metabolomics of BM plasma samples from patients with ITP and HDs. Plasma samples were stored at ‐80 °C after platelet removal for further metabolomics. E). Abundance differences of ALOX5 and PTGDS between ITP patient BM plasma samples and healthy donor BM plasma samples assessed by proteomic profiling. Data represent the mean or the mean with SD. *P* values: ns, *P* > 0.05; *, *P* < 0.05. F). The protein levels of cPLA2 and p‐cPLA2 were detected by western blotting. G). MIF reprograms AA metabolism by altering the expression patterns of ANXA1, PTGS2, and PTGES2, which are resistant to GC therapy. Dysfunctional AA metabolism affects platelet function and immunological homeostasis.

The expression of PTGS2 (COX‐2) can be influenced by transcription factors such as Sp1, Sp3, JUND, CREB1, JUN, and ATF6,^[^ ^32^ ^]^ suggesting that MIF is involved in AA metabolism. Moreover, MIF increased the protein levels of PTGS1 and EPHX2 (Figure 8D), providing concrete evidence for the MIF‐induced reprogramming of AA metabolism. Aberrant AA metabolism disrupts platelet activation and impaired immune regulation.^[^ ^33^ ^]^ Therefore, we investigated the expression of AA metabolism‐related genes following treatment with MIF and/or Dxm and found that MIF reduced *ANXA1* transcripts and inhibited Dxm‐induced ANXA1 expression (**Figure** **9**A).MIF Stimulation leads to the upregulation of *LTA4H* and *PTGES2* (Figure 9A; Figure S13A, Supporting Information), indicating the involvement of MIF in AA metabolism. Subsequently, the AA metabolism‐associated processes “KEGG_ARACHIDONIC_ACID_METABOLISM” and “REACTOME_ARACHIDONIC_ACID_METABOLISM” were evaluated, revealing a significant activation of AA metabolism specific to myeloid cells and T/NK (Figure S13B, Supporting Information). Alterations in AA metabolism have been observed in various cell types in patients with ITP (Figure S13C–F, Supporting Information). The increased expression of *ALOX5*, *LTA4H*, *TBXAS1*, *PTGES1*, *PTGS1*, and *PTGDS* transcripts in monocytes from patients with ITP confirmed heightened AA metabolic activity (Figure 9B). We performed mass spectrometry‐based proteomics and found that resistin and MIF induced different effects (Figure 9C). MIF treatment can upregulate the protein levels of CYP2U1. CYP2U1 is involved in the metabolism of arachidonic acid and its conjugates, which metabolize AA to the bioactive metabolites 19‐ and 20‐HETE.^[^ ^34^ ^]^ To test our hypothesis, we used oxidized fatty acid (OFAs) metabolomics to compare the levels of AA derivatives in the BM plasma of HDs and ITP patients. Consistent with the RNA data, elevated levels of lipid mediators in the plasma of patients with ITP also indicated enhanced PLA2 activity and AA metabolism (Figure 9D). Surprisingly, our study revealed that BM plasma samples from patients with ITP exhibited elevated PGD2 levels compared to those from HDs. PGD2 inhibits platelet aggregation, indicating that aberrant AA metabolism plays a role in ITP pathogenesis. Furthermore, our analysis identified increased levels of PGA2, an endogenous metabolite of PGE2, in the ITP samples. Our metabolomisc investigation revealed a previously unexplored library of metabolites implicated in ITP. Additionally, proteomic analysis of plasma samples revealed higher levels of the PTGDS protein in ITP samples than in HD samples, whereas the levels of ALOX5 were lower (Figure 9E; Figure S13G, Supporting Information). We also found that MIF treatment upregulated the phosphorylation of cPLA2 (Figure 9F), which contributes to AA metabolism. These results suggest that dysfunction of AA metabolism is involved in the pathogenesis of ITP (Figure 9G).

## 2 Discussion

Exosomes have been identified as vital biological mediators in the pathogenesis of ITP, with the upregulation of exosomal apolipoprotein (Apo) E observed in patients with ITP.^[^ ^35^ ^]^ Plasma ApoE is primarily synthesized by the liver, with contributions from macrophages and adipose tissue.^[^ ^36^ ^]^ Our findings demonstrated higher expression of exosomal MBL2, FCN2, and CFP proteins in BM plasma samples from patients with ITP than in those from HDs. MBL2, FCN2, and CFP are predominantly produced in the liver.^[^ ^37^ ^]^ The liver plays a role in platelet production by secretion of thrombopoietin (TPO).^[^ ^38^ ^]^ Our study identified a potential mechanism for the pathogenesis of ITP, indicating that abnormal activation of the liver‐associated lectin complement pathway may play a role in platelet destruction and immune responses facilitated by exosomes. This finding may provide insights into the development of thrombocytopenia in various conditions, including pregnancy, septic shock, pathogenic infections, autoimmune diseases, and vaccination, in which a clear pathogenesis is not readily apparent. Hepatitis viruses can induce immune responses through lectin‐dependent mechanisms, such as the interaction between hepatitis C virus (HCV)‐derived glycosylated envelope proteins E1 and E2 and MBL.^[^ ^39^ ^]^ Helicobacter pylori (H. pylori) infection may play a role in the development of ITP.^[^ ^40^ ^]^ Infection with H. pylori can affect the innate immune response in a lectin‐specific manner,^[^ ^41^ ^]^ and virulent strains of H. pylori stain can express mannans.^[^ ^42^ ^]^ Additionally, placenta and embryo‐derived exosomes may play a role in thrombocytopenia through dysfunctional immune tolerance during pregnancy.

Our scRNA‐seq analyses revealed the presence of a hyperactivated complement system in monocytes derived from patients with ITP. Decreased expression of CD55 in ITP monocytes suggests an imbalance in the complement system, which leads to heighten immune responses.^[^ ^43^ ^]^ Increased expression of C5AR1 in ITP monocytes suggests that C5A, a component of the complement system, amplifies inflammation. Our findings support the potential use of complement component 5 (C5) inhibitors such as ravulizumab, as a therapeutic option for ITP. In addition, targeting CD11b/CD11c‐mediated phagocytosis may offer an avenue for treatment.

The intricate interplay between resistin, MIF, and AA, as observed in patients with ITP, highlights the involvement of a complex regulatory network in the pathogenesis of this condition. The expression of MIF is associated with the clinical severity of various autoimmune diseases and is regulated by several key transcription factors such as ICBP90, Pit‐1, Sp1, and AP‐1.^[^ ^44^ ^]^ Additionally, elevated Sp1 levels can lead to the degradation of p53 through MDM2‐mediated ubiquitination.^[^ ^28a^ ^]^ Furthermore, TLR4 agonists have been shown to significantly increase MIF expression through ICBP90, prompting further investigation into the relationship between resistin and MIF. Our findings suggest a causal relationship between resistin and MIF, indicating the need for further investigation of resistin and MIF inhibitors as potential therapeutics for ITP. MIF activates cPLA2 through phosphorylation, thereby counteracting the GC‐mediated immunosuppression and promoting AA release.^[^ ^45^ ^]^ Additionally, AA has been found to downregulate resistin expression in 3T3‐L1 adipocytes in a manner dependent on COX‐1 (PTGS1) but not COX‐2 (PTGS2).^[^ ^46^ ^]^ MIF inhibits p53 activity in macrophages, leading to increased AA metabolism and COX‐2 expression.^[^ ^47^ ^]^ Furthermore, the inhibition of CD44 has demonstrated anti‐inflammatory properties in autoimmune disease models, such as ITP,^[^ ^48^ ^]^ suggesting that blocking the interaction between CD44/CD74 and MIF may also have anti‐inflammatory effects. Our research indicates that elevated levels of resistin and MIF in patients contribute to resistance to dxms treatment by competing with crucial signaling pathways.

A comprehensive understanding of the pathogenesis of ITP is essential for enhancing the clinical outcomes of thrombocytopenia and for the advancement of innovative immunotherapeutic approaches for autoimmune diseases, including ITP. Through proteomic of plasma and plasma‐derived exosomes from patients with ITP, our distinct dataset offers significant insights into complement system dysfunction, specifically highlighting the abnormal lectin pathway, CD11b/CD11c‐mediated phagocytosis, and downregulated CD55 and C5AR1‐mediated inflammation. Furthermore, our single‐cell analyses to verify biological functions using primary cells and cell lines, as well as metabolomic analyses, allowed us to uncover the gene regulatory mechanisms responsible for dysfunctional immune regulation. This underscores the significance of the resistin‐MIF‐AA axis in patients with ITP. In conclusion, our research offers enhanced insights into the molecular pathways implicated in the development of ITP and presents opportunities for the advancement of novel therapeutic approaches and the enhancement of current treatment modalities.

## 3 Experimental Section

### Specimen Collection

The study was carried out at The First Affiliated Hospital of Zhengzhou University (ZZU). Comprehensive data on the samples utilized for scRNA‐seq and other analyses are provided in Table S1. BMMCs and PBMCs were obtained through density gradient centrifugation. Blood and BM specimens from individuals with ITP and healthy volunteers were used in this study, which was approved by the Research and Clinical Trial Ethics Committee of First Affiliated Hospital of Zhengzhou University (2023‐KY‐0830‐002). All animal experiments were approved by the Ethical Committee of Zhengzhou University (ZZU‐LAC20210604[06]).

### Exosome Isolation and Characterization

Blood and BM plasma samples were collected, and cells, platelets, and dead cells were removed by centrifugation at 300 × g for 10 min and then 2000 × g for another 15 min. Cell debris was eliminated by centrifugation at 10 000 × g for 30 min. The samples were ultracentrifuged at 120 000 × g for 120 min. The exosomes were washed with phosphate‐buffered saline (PBS) again with the above conditions and re‐suspended in sterile PBS before storage at −80 °C for later use. The size distribution and concentration of exosomes were analyzed using nanoparticle tracking analysis (NTA) (ZetaView, Particle Metrix, Germany).

### NGL Mice Injected with BM Serum Exosomes

NGL mice were treated with 2 × 10^12^ BM exosomes from HDs and patients with ITP via the retro‐orbital injection (ROI). Twenty‐four hours later, the thymus, BM, spleen, PB, and lymph node were collected and processed into single‐cell suspensions for FCM analysis.

### Extraction of Exosome and Exosome‐Derived Proteins

After thawing the BM plasma samples on ice, they were centrifuged at 500 × g for 10 min at 4 °C to separate the supernatant, and remove dead cells or its derivatives. The supernatant was then subjected to a second centrifugation at 20 000 × g for 20 min at 4 °C to remove large vesicles such as cell debris and apoptotic bodies. Subsequently, the samples were transferred to ultracentrifuge tubes and centrifuged at 100 000 × g for 70 min at 4 °C. Following centrifugation, the supernatant was removed, and the pellet was resuspended in PBS. These processes required repetition. The resuspended pellet was used to collect high‐purity exosomes using a specific extraction kit. The collected exosomes were concentrated to a volume of 100 µL using 10KD ultrafiltration centrifuge tubes. Subsequently, the exosomes were transferred to 1.5 mL tubes and treated with 100µl TCEP mixtures containing protease inhibitors and phosphatase inhibitors. The proteins were denatured at high temperatures and cooled to room temperature. Next, an appropriate amount of trypsin was added for overnight digestion. The peptides were extracted, collected, dried at a low temperature, and reconstituted in 0.1% formic acid aqueous solution. The peptide solution was activated and equilibrated by sequential passage in acetonitrile and aqueous solutions. Following desalting, the peptide solution was dried and stored at ‐80 °C for subsequent analysis.

### Mass Spectrometry Data Collection of Exosome‐Derived Proteins

The samples were separated using a high‐performance liquid phase system (EASY‐cLC 1200) at nanoliter flow rates. Mobile phase A was a 0.1% formic acid aqueous solution and mobile phase B was a mixed solution of 0.1% formic acid and 80% acetonitrile. First, the chromatographic column was equilibrated with mobile phase A, and the enzymatic peptide fragments of the sample were transported to the loading column (2 cm, ID100 µm, 3 µm, C18) by the automatic sampler, and then passed through the analytical column (15 cm, ID150 µm, 1.9 µm, C18) at a flow rate of 600 nL min^−1^. Peptide samples separated from the chromatographic column were subjected to mass spectrometric analysis using an OE480 mass spectrometer. The detection mode was positive ion mode, the precursor ion scanning range was 300–1400m/z, the first‐level mass spectrometry resolution was 120 000 at 200m/z, the AGC (automatic gain control) target was 80 ms, the Maximum IT 80 ms, and the dynamic exclusion time was 40.0s. The mass‐to‐charge ratio of peptides and peptide fragments was collected according to the following method: the acquisition period of the fragment spectrum (MS2 scan) after each full scan was 1.5s, using HCD fragmentation mode, Normalized Collision Energy was 30%, and the isolation window is 1.6 m/z, and the secondary mass spectrometry resolution is 7500 at 200 m/z. The FAIMS voltages are set to ‐45V and ‐65V respectively, and other parameters are consistent.

### Exosomal Protein Identification, Quantification, and Analysis

RAW files were utilized as the primary source of data for analysis, and the iProteome one‐stop data analysis cloud platform was employed for both qualitative and quantitative analysis with identified sets (Enzyme: Trypsin; Fixed modifications: Carbamidomethyl (C); Variable modifications: Oxidation (M), Acetyl (Protein N‐term); Missed Cleavage: 2; Peptide Mass Tolerance: 20 ppm; Fragment Mass Tolerance: 0.05 Da, with the database being constructed by iProteome Co., Ltd). Differentially expressed proteins were annotated using *Blast2GO* and visualized using R package.

### Histology

Liver tissues that were fixed in formalin, embedded in paraffin, and sliced at a thickness of 5 µm were mounted onto slides. The sections were deparaffinized and dehydrated using gradient alcohol. Antigen retrieval buffer (Cat. No. G1203‐250ML, Servicebio) was applied to the sections, followed by treatment with 3% hydrogen peroxide at room temperature for 15 min in the dark. Subsequently, 3% BSA was added to the slides, which were sealed and incubated at room temperature for 30 min. Subsequently, slides were incubated with primary antibodies overnight at 4 °C, followed by three washes with PBS (pH 7.4) (Cat. No. G0002, Servicebio). The following primary antibodies were used: CD68 (D4B9C) XP Rabbit mAb (1:300, Cat. No. 76 437, CST), Anti‐CD31 Rabbit pAb (1:200, Cat. No. GB113151, Servicebio), Rabbit anti‐Ficolin 2 polyclonal antibody (1:100, Cat. No. ER1908‐87, HuaBio), MBL2 Polyclonal antibody (1:200, Cat. No. 24207‐1‐AP, proteintech), CFP Polyclonal antibody (1:200, Cat. No. 17192‐1‐AP, proteintech), and Albumin Polyclonal antibody (1:400, Cat. No. 16475‐1‐AP, proteintech). The slides were incubated with secondary antibodies specific to the primary antibody's species at room temperature for 50 min. The following secondary antibody was used: goat anti‐mouse/rabbit IgG H&L (Cat. No. RCB054, RecordBio). After the slides were washed three times with PBS, they were exposed to tyramide a seven‐color multiple immunofluorescence kit (Recordbio Biological Technology, Shanghai, China) based on the tyramide signal amplification (TSA) technology according to the manufacturer's instructions (Cat. No. RC0086‐67RM, RecordBio). Cell nuclei were visualized using DAPI (Cat. No. G1012, Servicebio).

### Concanavalin A (ConA) Mouse Model

Female C57BL/6 Rosa26‐mTmG (Rosa26‐loxP‐STOP‐loxP‐mT‐pA‐loxP‐STOP‐loxP‐mG) mice, aged 8 to 12 weeks and weighing between 20 to 22 grams, were used to establish a Concanavalin A (ConA)‐induced murine model. The mice were randomly assigned to two groups: (1) the negative control (NC) group, which received an intravenous injection of DPBS and (2) the ConA‐induced autoimmune hepatitis (AIH) group, which received ConA (Catalog No. S12028‐25mg, Shanghai Yuanye Bio‐Technology Co., Ltd) intravenously at a dose of 20 mg kg^−1^ body weight. All mice were euthanized at six hours post‐injection for subsequent sample collection.

### Carrageenan‐Induced Tail Thrombosis (CTT)

NGL mice were administered 1 × 10^13^ BM exosomes from HDs and patients with ITP via the ROI. Two hours after administration, carrageenan was administered at a dose of 100 mg kg^−1^. Subsequently, carrageenan was administered at the same dose 24 h later. Venous thrombus formation was evaluated 24 h after carrageenan administration. For in vivo bioluminescence imaging, mice were immediately placed under isoflurane anesthesia (2‐3%), and images of the abdomen or back were captured using an IVIS Spectrum system. Radiance was quantified in photons per second using integrated Living Image software. Organ and tissue samples obtained from mice were measured using the IVIS Spectrum system and subsequently fixed with 4% PFA for hematoxylin and eosin (H&E) staining.

### Gene Expression Analysis using Quantitative Real‐Time PCR (qRT‐PCR)

Total RNA was isolated with TRIzol reagent (Cat. No. 15 596 026, Invitrogen), followed by the generation of cDNA libraries through reverse transcription using the Hifair AdvanceFast One‐step RT‐gDNA Digestion SuperMix for qPCR (Cat. No. 11151ES60, YEASEN). Quantitative PCR was conducted using the Hieff qPCR SYBR Green Master Mix (Low Rox Plus) (Cat. No. 11202ES08, YEASEN) according to the manufacturer's guidelines. The primer sequences used in this study are listed in Table S2. BMMCs were isolated using Ficoll‐Paque PLUS and cultured in heat‐inactivated or untreated autologous plasma. rhMBL‐2(Cat. No. C488, Novoprotein), rhFicolin‐2 (Cat. No. 2428‐FC, R&D Systems), and rhProperdin (Cat. No. 8216‐PR‐050, R&D Systems) proteins were added to the complete medium for 24h to investigate changes in the selected genes using qRT‐PCR.

### Assay for Transposase‐Accessible Chromatin with High‐Throughput Sequencing (ATAC‐Seq)

PBMCs were cultured in complete RPMI 1640 medium with or without Recombinant Human MIF (200 µg mL^−1^, Cat. No. CH33, novoprotein) for 12 h before being use in ATAC‐seq. Library amplification was performed according to the manufacturer's instructions using a High‐Sensitivity Open Chromatin Profile Kit 2.0 (for illumina) (Cat. No. N248, Novoprotein). The adapters (N702: CGTACTAG and N502: CTCTCTAT) were removed from the reads using Cutadapt. Paired‐end alignment was conducted using a bowtie, allowing for a maximum distance of 2 kb between mates. Peak identification was performed using the *findPeaks* command of HOMER.

### Flow Cytometry

PBMC and BM mononuclear cells were isolated using Ficoll‐Paque PLUS (Cat. No. 17 144 003, Cytiva) as previously described.^[^ ^49^ ^]^ Cells were incubated with different combinations of antibody mixtures (PE anti‐human CD284 (TLR4) Antibody (Clone No. HTA125, Cat. No. 312 805, BioLegend), APC Anti‐Human CD14 Antibody (Clone No. M5E2, Cat. No. E‐AB‐F1209E, Elabscience), APC/Cyanine7 anti‐human CD16 Antibody (Clone No. 3G8, Cat. No. 302 018, BioLegend), PE Anti‐Human CD16 Antibody (Clone No. 3G8, Cat. No. E‐AB‐F1236D, Elabscience), APC/Cyanine7 anti‐human CD46 Antibody (Clone No. TRA‐2‐10, Cat. No. 352 410, BioLegend), PE/Cyanine7 anti‐human CD88 (C5aR) Antibody (Clone No. S5/1, Cat. No. 344 308, BioLegend), Biotin anti‐human CD35 Antibody (Clone No. E11, Cat. No. 333 414, BioLegend), FITC anti‐human CD55 Antibody (Clone No. JS11, Cat. 311 306, BioLegend), PE anti‐human CD59 Antibody (Clone No. p282 (H19), Cat. 304 708, BioLegend), PerCP/Cyanine5.5 anti‐mouse/human CD11b Antibody (Clone No. M1/70, Cat. No. 101 228, BioLegend)), Biotin anti‐human CD11c Antibody (Clone No. Bu15, Cat. No. 337 232, BioLegend), APC anti‐human C3AR Antibody (Clone No. hC3aRZ8, Cat. No. 345 806, BioLegend), FITC anti‐human CD18 Antibody (Clone No. TS1/18, Cat. No. 302 106, BioLegend), Biotin anti‐human/mouse CXCR7 Antibody (Clone No. 8F11‐M16, Cat. No. 331 111, BioLegend), APC/Cyanine7 anti‐mouse/human CD44 Antibody (Clone No. IM7, Cat. No. 103 028, BioLegend), PE anti‐human CD184 (CXCR4) Recombinant Antibody (Clone No. QA18A64, Cat. No. 304 504, BioLegend), APC anti‐human CD74 Antibody (Clone No. LN2, Cat. No. 326 812, BioLegend) for 45 min in the dark at 4 °C. PE‐streptavidin (Cat. No. 405 204, BioLegend) was used in some of the experiments. DAPI and the Zombie NIR Fixable Viability Kit (Cat. No. 423 106, BioLegend) were used to eliminate interference from dead cells. Samples were strained through 70µm mesh, and analyzed with Aria II.

### Cells Stimulated by Pseudovirus

THP‐1 cells were maintained in RPMI‐1640 medium (Cat. No. G4531‐500ML, Servicebio) supplemented with 10% fetal bovine serum (FBS) (Cat. No. FSS500, excell) and 0.05 mM 2‐mercaptoethanol (Cat. No. M3148‐25ML, sigma). Upon activation, THP‐1 cells were cultured in RPMI‐1640 medium supplemented with 10% FBS and stimulated with 100 ng/mL PMA (Cat. No. s1819, beyotime) for 24 h. PBMCs, and HepG2 cells were cultured in DMEM medium (Cat. No. 10‐013‐CV, CORNING) supplemented with 10% FBS. Cells were seeded at a density of 1×10^5^ per well in a 6‐well plate with 2 mL of culture medium and incubated overnight. Subsequently, the novel coronavirus N‐gene pseudovirus (Cat. No. C3201, Beyotime) was added to each well at concentrations of 0, 0.02, 0.2, and 1. Cells were cultured for an additional 48h, harvested by centrifugation at 300 × g for 5 min, and washed twice with PBS. Total RNA was extracted using TRIZOL reagent for subsequent quantitative polymerase chain reaction (qPCR) analysis following established protocols.^[^ ^50^ ^]^

### Cells Cultured with Exosomes

BMMCs from healthy donors were isolated using Ficoll‐Paque reagent, and erythrocytes were removed by treatment with red blood cell (RBC) lysis buffer (Cat. No. R1010, Solarbio). The cells were washed twice with PBS, resuspended in RPMI1640 medium, and seeded into individual wells of a 24‐well plate at a density of 1×10^6^ cells mL^−1^. Exosomes were labeled with CFSE (Cat. No. FMS‐CF‐500, FCMACS) in plasma and isolated using the Exosome Extraction & Purification Kit (Cat. No. DL21010, Duolaimi Biotechnology), according to the manufacturer's instructions. The exosomes were quantified using a BCA kit (Cat. No. ZJ101, Rpizyme Biomed), and then added to PBMCs from BMMCs at a concentration of 100 ug per well. Following a 24‐h incubation peripheral cells were collected, fixed with 4% PFA (Cat. No. G1101‐500ML, servicebio), and resuspended in PBS. CFSE‐stained BMMCs were analyzed using FCM, and the data were processed using FlowJo software.

### Cells Cultured with Platelet and Exosomes

PBMCs, serum, and platelets were isolated from HDs using a previously described method.^[^ ^51^ ^]^ The platelets were then labeled with DIL dye (Cat. No. G1705, Servicebio.) according to the manufacturer's protocol, washed with platelet' wash buffer, and resuspended in pre‐warmed HEPES‐modified Tyrode's buffer. Exosomes were stained with CFSE (Cat. No. FMS‐CF, FcMacs Biotech.), harvested using an Exosome Extraction and Purification Kit (Cat. No. DL21010, Duolaimi Biotechnology), and quantified using a BCA Kit (Cat. No. ZJ101, Epizyme Biomed). The cells were resuspended in RPMI 1640, and seeded into each well of a 24‐well plate at a density of 5 × 10^5^ cells mL^−1^, co‐cultured with 30 µg of exosomes, 5 × 10^7^ platelets, and 25 µL of serum, either heat‐inactivated or not, at 56 °C for 30 min. Following 24h of incubation, cells were collected and washed twice with PBS. Subsequently, the cells were stained with APC Anti‐Human CD14 Antibody (Clone No. M5E2, Cat. No. E‐AB‐F1209E, Elabscience) for 45 min on ice after blocking with an Fc receptor‐blocking solution for 15 min. The cells were washed twice with wash buffer and stained with DAPI. A BD cytometer was used for the analysis, and the data were analyzed using the FlowJo software.

### Cells Cultured with Mannan‐Coated Platelets

Platelets were obtained by centrifugation at 460×g for 17 min without a brake, and the whole blood supernatant was centrifuged at 200×g for 5 min. PBMCs were isolated using the Ficoll‐Paque reagent following the aforementioned method. Human serum was collected from the supernatant of the red‐capped tubes after centrifugation at 12 000×g for 15 min from the same HD. Initially, a volume of 100 µL of platelets (1× 10^8^) was subjected to incubation with 1µg of mannan (Cat. No. M7504, Sigma) at 37 °C for 1h in pre‐warmed HEPES‐modified Tyrode's buffer. Subsequently, the platelets, mannan, or the pre‐incubated Platelet‐mannan mixture was introduced to the PBMCs and incubated for 30 min at 37 °C with 25µL of human serum, which had either not been treated or had undergone heat inactivation (56 °C for 30min). The incubation process was halted by the addition of 50 µL of 20 mM EDTA buffer, after which the samples were prepared for FCM analysis.

### Enzyme‑Linked Immunosorbent Assay (ELISA) Assay

Blood and BM plasma samples underwent centrifugation at 2000 × g for 20 min at 4 °C to eliminate platelets, with the resulting supernatant then being preserved at ‐80 °C for subsequent analysis. These samples were subsequently analyzed using ELISA for quantification of human FCN2 (Cat. No. ELK2672, ELK Biotechnology), MBL2 (Cat. No. ELK1098, ELK Biotechnology), CFP (Cat. No. ELK3011, ELK Biotechnology), MIF (Cat. No. ELK1506, ELK Biotechnology), RETN (Cat. No. ELK1225, ELK Biotechnology), and CXCL12 (Cat. No. EK1119, MULTI SCIENCES).

PBMCs were collected and resuspended in the culture medium at a concentration of 1 × 10^8^ cells mL^−1^. The cells were treated with FC blocking buffer for 15 min on ice in the dark. Subsequently, B cells were isolated using the easySep human CD19 positive selection kit II (Cat. No. 17 854, Stemcell tech.), following the manufacturer's instructions. Isolated cells were seeded at a concentration of 1 × 10^6^ cells mL^−1^ and exposed to resistin (100ng mL^−1^) for 24 h before harvesting by centrifugation at 400 × g for 5 min. Proteins were extracted using RIPA lysis buffer supplemented with a protease inhibitor cocktail (Cat. No. HY‐K0010, MCE) and phosphatase inhibitor cocktail I (HY‐K0021, MCE), quantified using the BCA assay, and normalized with lysis buffer. TSC22D3 quantification was subsequently conducted using an ELISA kit (Cat. No. ELK1946, ELK Biotechnology) according to the manufacturer's protocol.

### Western Blotting

Western blot analysis was performed, and the results were interpreted in accordance with the manufacturer's instructions. THP‐1 cells, at a concentration of 1 × 10^6^ were initially treated with 100ng/mL PMA (Cat. No. S1819, Beyotime) for 24 h, followed by a 24‐h incubation with medium replacement. Subsequently, the cells were exposed to 100 ng resistin for 30 min before harvesting. Nuclear and cytoplasmic proteins were isolated using a nuclear and cytoplasmic protein extraction kit (Cat. No. P0027, Beyotime) according to the manufacturer's instructions. In some cases, the samples were directly lysed in RIPA buffer (Cat. No. B001, kmtbio) containing a Protease Inhibitor Cocktail (Cat. No. HY‐K0010, MCE) or Phosphatase Inhibitor Cocktail II (Cat. No. HY‐K0022, MCE) for 30 min. Proteins were quantified using a BCA assay, normalized with lysis buffer, denatured before being separated on 10% SDS‐PAGE gels (Cat. No. PG212, Epizyme), and transferred onto PVDF membranes. The proteins were then incubated overnight at 4 °C with specific antibodies, including Phospho‐NF‐κB p65 (Ser536) (93H1) Rabbit mAb (Cat. No. 3033, CST), GAPDH Monoclonal antibody (Cat. No. 60004‐1‐Ig, proteintech), and Anti‐NF‐kB p65 Rabbit pAb (1:100, Cat. No. GB11997‐100, servicebio). Beta Actin Monoclonal antibody (Cat. No. Cat No. 66009‐1‐Ig, proteintech), cPLA2 (D49A7) Rabbit mAb (Cat. No. 5249S, Cell Signaling), and Phospho‐PLA2G4A (Ser505) Polyclonal antibody (Cat. No. 28925‐1‐AP, proteintech) were used for detecting the expression of cPLA2 and its phosphorylation after MIF treatment. Secondary antibodies labeled with horseradish peroxidase and the Omni‐ECL Femto Light Chemiluminescence Kit (Cat. No. SQ201, EpiZyme) were used for visualization using a gel imaging system (Amersham imager 600, GE). Immunoreactive bands were quantified using ImageJ software.

### Mass Spectrometry of OFAs Metabolic Profiles

Five BM plasma samples devoid of blood cells and platelets from HDs andnine samples from patients with ITP, were used to generate oxidized fatty acid (OFAs) metabolic profiles (Wuhan Metware Biotechnology Co., Ltd). The specific experimental procedures have been described in our previous report.^[^ ^49^ ^]^

### Proteome Analysis of Plasma Samples

BM plasma samples were depleted of cells and platelets, then stored at ‐80 °C. The thawed samples were mixed with 98 µL of 50 mM ammonium bicarbonate solution (Cat. No. A6141‐1KG, Sigma‐Aldrich), heated at 95 °C for 3 min for protein denaturation, and subsequently cooled to room temperature. Trypsin was used to digest the samples at an enzyme/protein mass ratio of 1:25 overnight at 37 °C, followed by extraction and drying of the peptides using a Speedvac (Eppendorf). The peptides were reconstituted in 0.1% formic acid solution and subsequently identified using mass spectrometry (MS).

For proteome profiling, peptides were analyzed using a Q Exactive HF‐X Hybrid Quadrupole‐Orbitrap Mass Spectrometer (Thermo Fisher Scientific) in conjunction with a high‐performance liquid chromatography system (EASY nLC 1200, Thermo Fisher Scientific). The dried peptide samples were re‐dissolved in Solvent A (0.1% formic acid in water) (Cat. No. A117‐50, Fisher) and subsequently loaded onto a 2‐cm self‐packed trap column (100 µm inner diameter, 3 µm ReproSil‐Pur C18‐AQ beads, Dr. Maisch GmbH) using Solvent A. The samples were then separated on a 150‐µm‐inner‐diameter column with a length of 15 cm (1.9 µm ReproSil‐Pur C18‐AQ beads, Dr. Maisch GmbH). The eluted peptides were ionized at 2 kV and introduced into a mass spectrometer for analysis in a data‐independent manner (DIA). The DIA method employed in this study involved conducting MS1 scans within the range of 300–1400 m/z in a solution of 60000 (AGC target 4e5 or 50 ms). Subsequently, 30 DIA segments were captured at a resolution of 15000 with an AGC target of 5e4 or 22 ms for the maximal injection time. HCD fragmentation was applied with a normalized collision energy of 27%, and the spectra were acquired in the profile mode. The default charge state of MS2 is designated as 3.

RAW files served as the raw data for subsequent analysis, and the iProteome one‐stop data analysis cloud platform was utilized for both qualitative and quantitative analysis of the identified sets (Enzyme: Trypsin; Fixed modifications: Carbamidomethyl (C); Variable modifications: Oxidation (M), Acetyl (Protein N‐term); Missed Cleavage: 2; Peptide Mass Tolerance: 20 ppm; Fragment Mass Tolerance: 0.05 Da, database was constructed by iProteome Co., Ltd). Differentially expressed proteins were annotated utilizing Blast2GO and visualized using the R package.

### Quantitative Proteomics of Cytokine‐Treated Cells

THP‐1 cells were cultured in RPMI‐1640 medium supplemented with 10% FBS and stimulated with 100 ng mL^−1^ PMA for 24 h. Then these PMA‐treated THP‐1 cells were treated with resistin or MIF for 24h and stored at ‐80 °C freezer. The thawed samples were treated with a lysis buffer (8M urea, 1mM PMSF, and 2mM EDTA) and subjected to ultrasonic lysis for 5 min on ice for 10 min. Then the samples were centrifuged at 15 000g at 4 °C for 10 min to collect the supernatant and identified the protein concentration using BCA assay kit. Proteolytic desalting, LC‐MS/MS analysis, database search, and quantification are described in a previous publication.

### Single‐Cell Library Construction and Sequencing

Single‐cell suspensions in PBS at a concentration of 100 000 cells mL^−1^ were prepared and loaded onto the microfluidic devices using at Singleron Matrix Single Cell Processing System. The scRNA‐seq libraries were prepared following the protocol outlined in the GEXSCOPE Single‐Cell RNA Library Kit (Cat. No. 5 180 012, Singleron), with individual libraries diluted and pooled for sequencing on an Illumina HiSeq X system utilizing 150‐bp paired‐end reads. The initial analysis of the raw read data can be found in our previous publication.^[^ ^49^ ^]^

### Quality Control, Dimension Reduction, Clustering, and Differentially Expressed Gene (DEG) Analysis

Uniform Manifold Approximation and Projection (UMAP) was employed for dimensionality reduction using of Seurat, while the Harmony package was utilized for the integration of data from multiple samples to mitigate batch effects. Various cell types were distinguished based on their unique expression patterns, including CD14^+^CD16^−^ Monocytes expressing *S100A8*, *S100A9*, *S100A12*, *CD14*, *ITGAM*, and *LYZ*; CD16^+^ Monocytes expressing *FCGR3A*, *CDKN1C*, and *MS4A*; erythroid lineage expressing *HBA1*, *HBA2*, *HBB*, *GYPA*, *KLF1*, and *GATA1*; HSPC expressing *MYB*, *GATA2*, *CD34*, and *KIT*; T/NK expressing *CD3D*, *CD3E*, *CD3G*, *TRAC*, *IL7R*, *KLRD1*, *NKG7*, and *CST7*; DC expressing *CD1C*, *HLA*‐*DPB1*, *HLA*‐*DPA1*, *HLA*‐*DQA1*, *FCER1A*, and *CLEC10A*; B cells expressing *CD19*, *MS4A1*, *IGHD*, *IGHM*, *IL4R*, and *TCL1A*; plasma cell expressing *CD27*, *CD38*, *XBP1*, *MZB1*, and *SDC1*; pDC expressing *IL3RA*, *JCHAIN*, *IRF7*, *TCF4*, *LILRA4*, and *CLEC4C*; stromal cells expressing *VCAN*, *C7*, *FN1*, and *FBN1*. Different subsets of pDC and myeloid cells were categorized based on their specific expression patterns, including markers such as *CSF1R*, *CX3CR1*, *CD68*, *CD86*, *C1AQ1*, *C1QB*, *C1QC*, *ADGRE1*, *MERTK*, *CD40*, *MARCO*, and *TNF* in macrophages; *IL3RA*, *JCHAIN*, *IRF7*, *TCF4*, *LILRA4*, and *CLEC4C* in pDCs; *CD1C*, *HLA*‐*DPB1*, *HLA*‐*DPA1*, *HLA*‐*DQA1*, *FCER1A*, *CLEC10A*, and *FCGR2B* in DCs; *CD14*, *VCAN*, *S100A8*, *S100A9*, *LYZ*, and *S100A12* in monocytes. Dimension reduction plots (UMAP), heatmaps, violin plots, dot plots, and feature plots were generated using various functions in Seurat, such as DimPlot, DoHeatmap, VlnPlot, DotPlot, and FeaturePlot. The cells were assessed for gene expression using the *addModuleScore* function in Seurat. GO analysis was performed using *clusterProfiler* package. The CellChat R package was used to explore cellular interactions between different immune cell subsets. The resulting enriched interaction network was visually represented using chord diagrams, in which the thickness of the arcs reflected the overall strength of the interactions.

### Statistical Analysis

All the data in this study were presented as mean ± standard deviation (SD) and analyzed utilizing two‐tailed unpaired Student's t‐test for identifying the difference between two groups. All statistical analyses were performed using Graphpad Prism 9.5.0. A P value of less than 0.05 was considered statistically significant. Statistical analyses and graphing were performed using the R software with the R package *ggplot2*.

### Data Availability

The scRNA‐seq data supporting the conclusions of this study have been archived in the National Genomics Data Center (NGDC), Genome Sequence Archive (GSA) database under the accession of HRA007854 (patients with ITP) and HRA005145 (HDs). The Cut&Tag sequence data reported in this paper have been deposited in the NDGC, GSA database under the accession of HRA008295 that are publicly accessible at https://ngdc.cncb.ac.cn/gsa‐human. All other data supporting the findings of this study are available from the corresponding author on reasonable request. The bulk RNA sequence data reported in this paper have been deposited in the NDGC, GSA database under the accession of HRA008338 that are publicly accessible at https://ngdc.cncb.ac.cn/gsa‐human. The Other data or further information could be obtained from Rongqun Guo (guorq2007@163.com or fccguorq@zzu.edu.cn).

## Conflict of Interest

The authors declare no conflict of interest.

## Author Contributions

J.L., X.W., Y.C., X.S., and L.F. contributed equally to this work. J.L.: data curation, investigation, validation; X.W.: Data curation, investigation, validation; Y.C.: formal analysis, investigation, validation; X.S.: data curation, validation, visualization; L.F.: data curation, formal analysis, validation; Q.X.: validation; H.Z.: validation; B.Q.: conceptualization; N.S.: validation; Y.L.: resources; Y.X.: resources; H.Y.: validation; D.H.: validation; Y.D.: validation; S.W.: validation; M.Z.: validation; Q.L.: validation; F.W.: conceptualization, supervision, writing; B.Y.: project administration, supervision, writing‐review & editing; Y.G.: conceptualization, funding acquisition, project administration, resources; Y.J.: conceptualization, funding acquisition, project administration, supervision, writing‐review & editing; R.G.: conceptualization, data curation, formal analysis, funding acquisition, investigation, methodology, project administration, Resources, software, supervision, validation, visualization, writing‐original draft, writing‐review & editing.

## Supporting information



Supporting Information

## Data Availability

The data that support the findings of this study are available from the corresponding author upon reasonable request.
