# Supplementary material for: Exosome‐Mediated Lectin Pathway and Resistin‐MIF‐AA Metabolism Axis Drive Immune Dysfunction in Immune Thrombocytopenia
Source: Adv Sci (Weinh). 2025 Jan 10;12(10):2412378. doi: 10.1002/advs.202412378 (PMC11905005; doi:10.1002/advs.202412378)
Supplement: Supplementary file 1 — Supporting Information [file ADVS-12-2412378-s001.docx]

**Supporting Information**

**Title: Exosome-mediated Lectin Pathway and Resistin-MIF-AA Metabolism Axis Drive Immune Dysfunction in Immune Thrombocytopenia**

**Authors:** *Jin Li*^*^, *Xiaoqian Wang*^*^, *Yaoyao Chen*^*^, *Xianlei Sun*^*^, *Liyan Fu*^*^, *Qingxuan Xin*, *Huilin Zhang*, *Bo Qin*, *Nannan Sun*, *Yingmei Li*, *Yan Xu*, *Hui Yang*, *Dawei Huo*, *Yong Dong*, *Shuya Wang*, *Mengyun Zhao*, *Quande Lin*, *Fang Wang*^#^, *Baohong Yue*^#^, *Yanxia Gao*^#^, *Yong Jiang*^#^, *Rongqun Guo*^#^


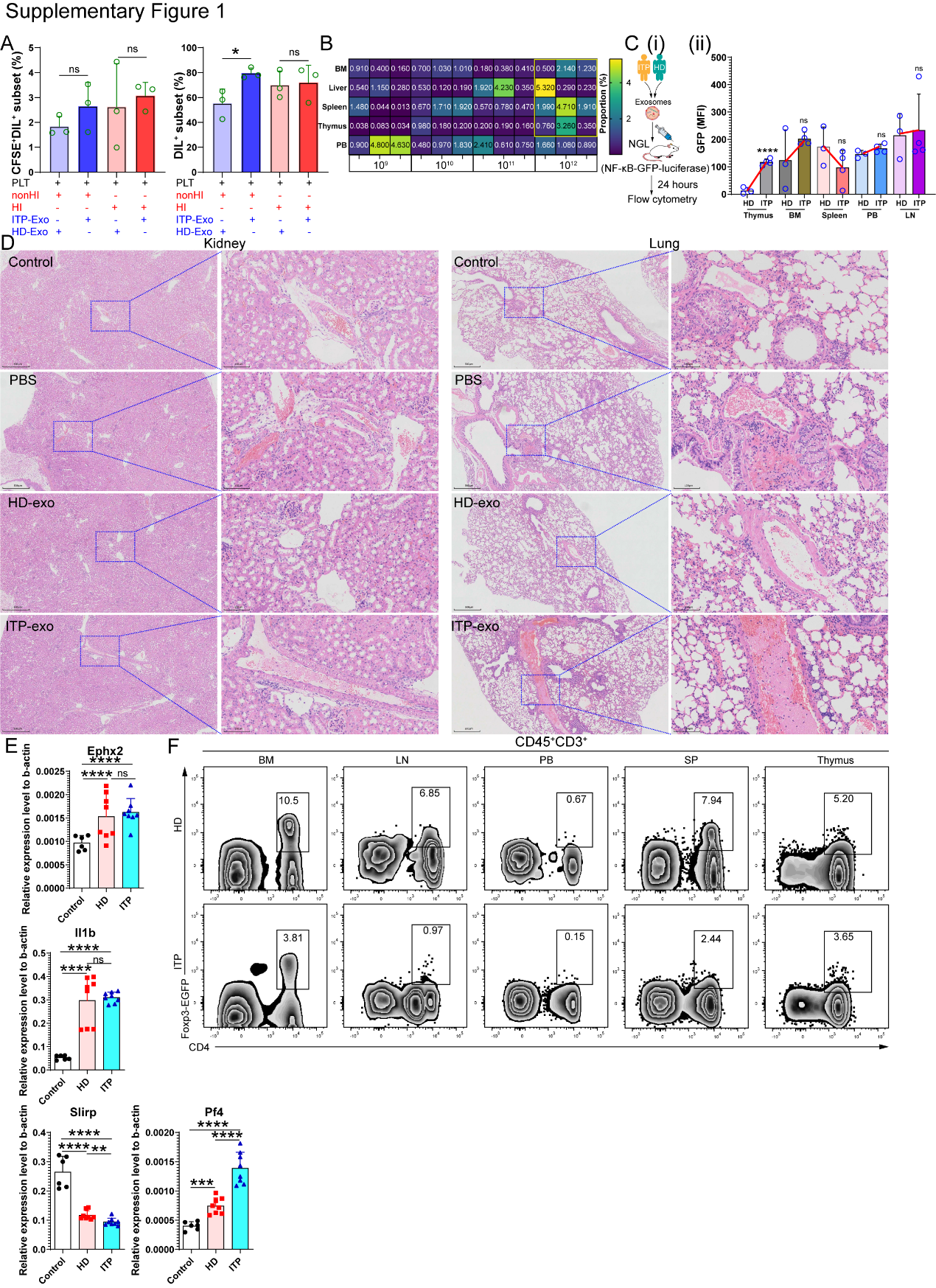


**Figure S1. Exosomes from patients with ITP promote platelet destruction.**

**(A).** Bar graphs illustrating the percentages of CFSE-labeled and CFSE/DIL-labeled cells measured by flow cytometric quantification of CD14^+^ monocytes. *P* value significance represented by *, <0.05; **, <0.01; ***, <0.001; ****, <0.0001.

**(B).** The proportion of DIL^+^ cells in different tissues (BM, liver, spleen, thymus, and PB) after 24h treatment with different concentrations of DIL-labeled exosomes.

**(C).** Experimental methodology for examining exosome-induced NF-κB activation. Summary graphs showing median fluorescence intensity (MFI) of GFP in cells of different tissues (thymus, BM, spleen, PB, and lymph node [LN]) from NGL mice treated with ITP-exosomes and HD-exosomes. *P* value significance represented by *, <0.05; **, <0.01; ***, <0.001; ****, <0.0001.

**(D).** H&E staining of kidneys and lungs from mouse with carrageenan infusion after treatment with exosomes from ITP patients and healthy donors.

**(E).** Quantification of selected genes mRNA expression in BM cells from the control (n=6), HD-exo-treated (n=8), and ITP-exo-treated groups (n=8)**.** *P* value significance represented by *, <0.05; **, <0.01; ***, <0.001; ****, <0.0001.

**(F).** Flow cytometry analysis of immune cells in the BM, LN, PB, SP, and thymus based on the detection of CD4^+^FoxP3^+^ Treg-like cells. Representative plots are shown.


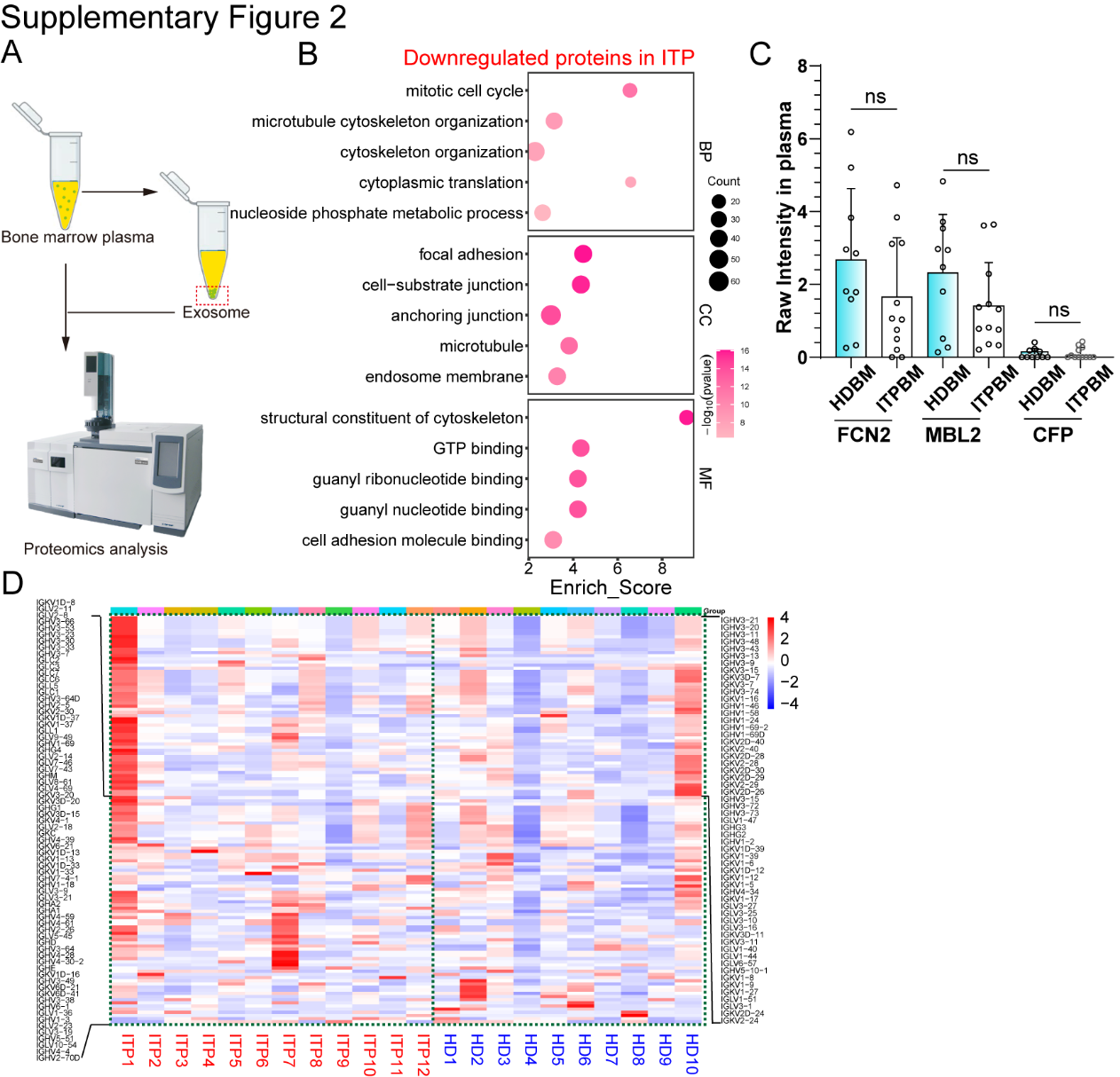


**Figure S2. Lectin pathway-specific exosome is a remarkable feature of BM plasma from patients with ITP.**

**(A).** Experimental workflow.

**(B).** Gene Ontology (GO) enrichment analysis of downregulated exosomal proteins in patients with ITP. BP, biological process; CC, cellular component; MF, molecular function.

**(C).** Abundance differences of FCN2, MBL2, and CFP between ITP patients BM plasma samples and healthy donor BM plasma samples assessed by proteomic profiling. Data represent the mean or the mean with SD. P values: ns, P ＞0.05.

**(D).** Heatmap showing the normalized expression of immunoglobulin in BM plasma samples of ITP patients (n=12) and HDs (n=10).


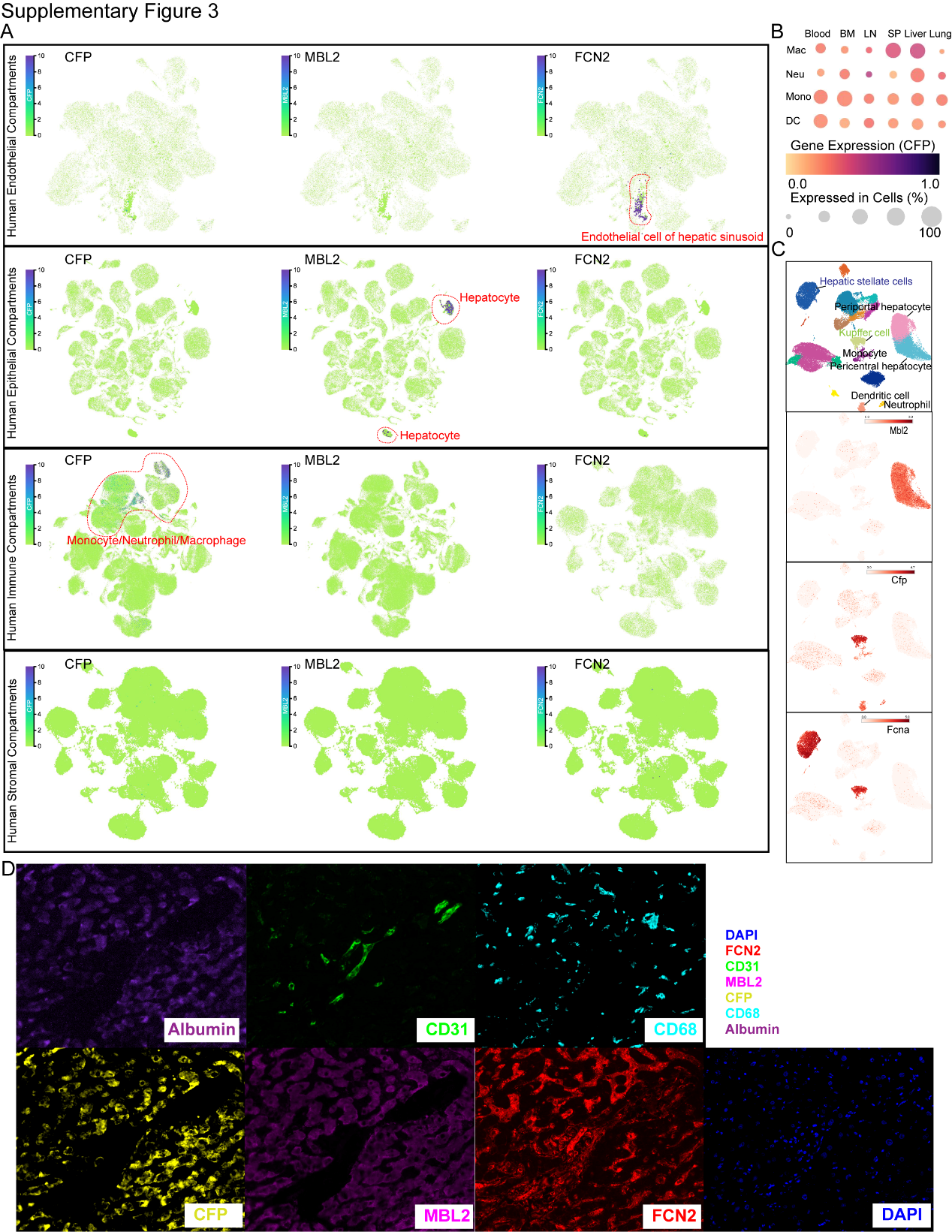


**Figure S3. Liver-associated cell populations are potential sources of MBL2, FCN2, and CFP.**

**(A).** The expression levels of CFP, MBL2, and FCN2 in human endothelial, epithelial, immune, and stromal compartments from a human multiple organ single cell transcriptomic atlas (named “Tabula Sapiens,” https://tabula-sapiens-portal.ds.czbiohub.org/) including 24 organs of 15 normal human subjects^1^.

**(B).** Dot plots showing CFP expression in different myeloid subsets (macrophages, neutrophils, monocytes, and DC) from predicted organs or tissues including blood, bone BM, lymph node (LN), spleen (SP), liver, and lungs. CFP expression patterns were obtained from a dataset derived from “Tabula Sapiens” (https://tabula-sapiens-portal.ds.czbiohub.org/).

**(C).** UMAP plots showing the expression patterns of *Cfp*, *Mbl2*, and *Fcna* (resembling human *FCN2*) in a single cell transcriptome atlas of the mouse liver (https://db.cngb.org/stomics/lista/scRNA.seq/)^2^.

**(D).** Immunofluorescence staining of tumor-adjacent liver tissue from patients with human hepatocellular carcinoma (HCC). Each fluorescent panel is presented alone.


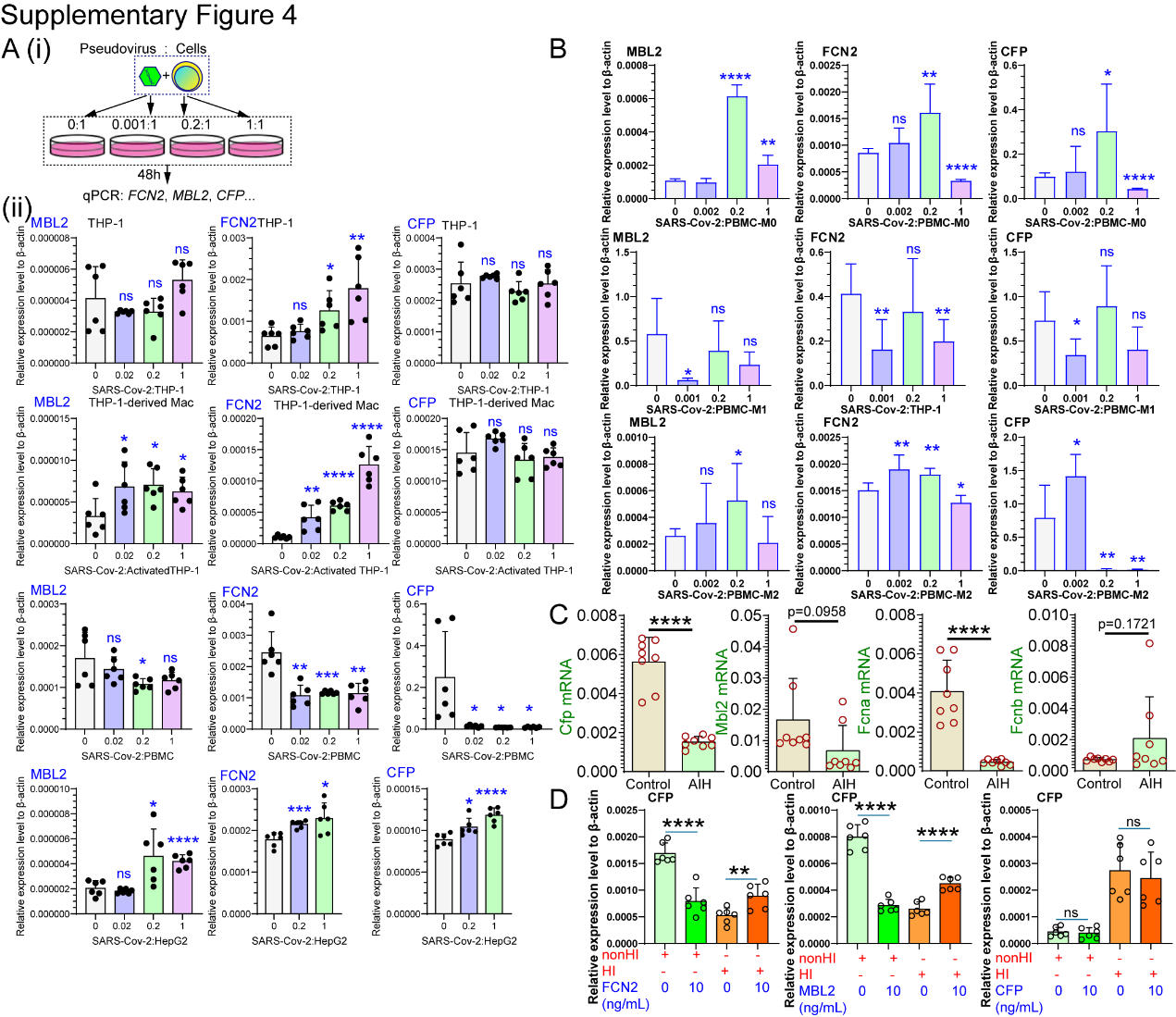


**Figure S4. Pathogens were identified as a mediator of expression of CFP, FCN2, and MBL2 in some cell types.**

**(A).** Experimental timeline (i). Quantification of *FCN2*, *MBL2*, and *CFP* mRNA expression in control and pseudovirus-treated groups (ii). Several cell types, including THP-1 cells, THP-1-derived M0 macrophages, PBMCs, and HepG2 cells, were examined after different treatments. *P* value significance represented by *, <0.05; **, <0.01; ***, <0.001; ****, <0.0001.

**(B).** Quantification of *FCN2*, *MBL2*, and *CFP* mRNA expression in the control and pseudovirus-treated groups (PBMC-derived M0, M1, and M2). *P* value significance represented by *, <0.05; **, <0.01; ***, <0.001; ****, <0.0001.

**(C).** Quantification of *Mbl2*, *Cfp*, *Fcna*, and *Fcnb* mRNA expression in the livers of WT mice treated with normal liver-derived exosomes (n=8) or AIH-exosomes (n=8). *P* value significance represented by *, <0.05; **, <0.01; ***, <0.001; ****, <0.0001.

**(D).** *CFP* transcript levels were estimated after MBL2, FCN2, and CFP stimulation in BMMCs from HDs cultured with heat-inactivated or normal plasma.


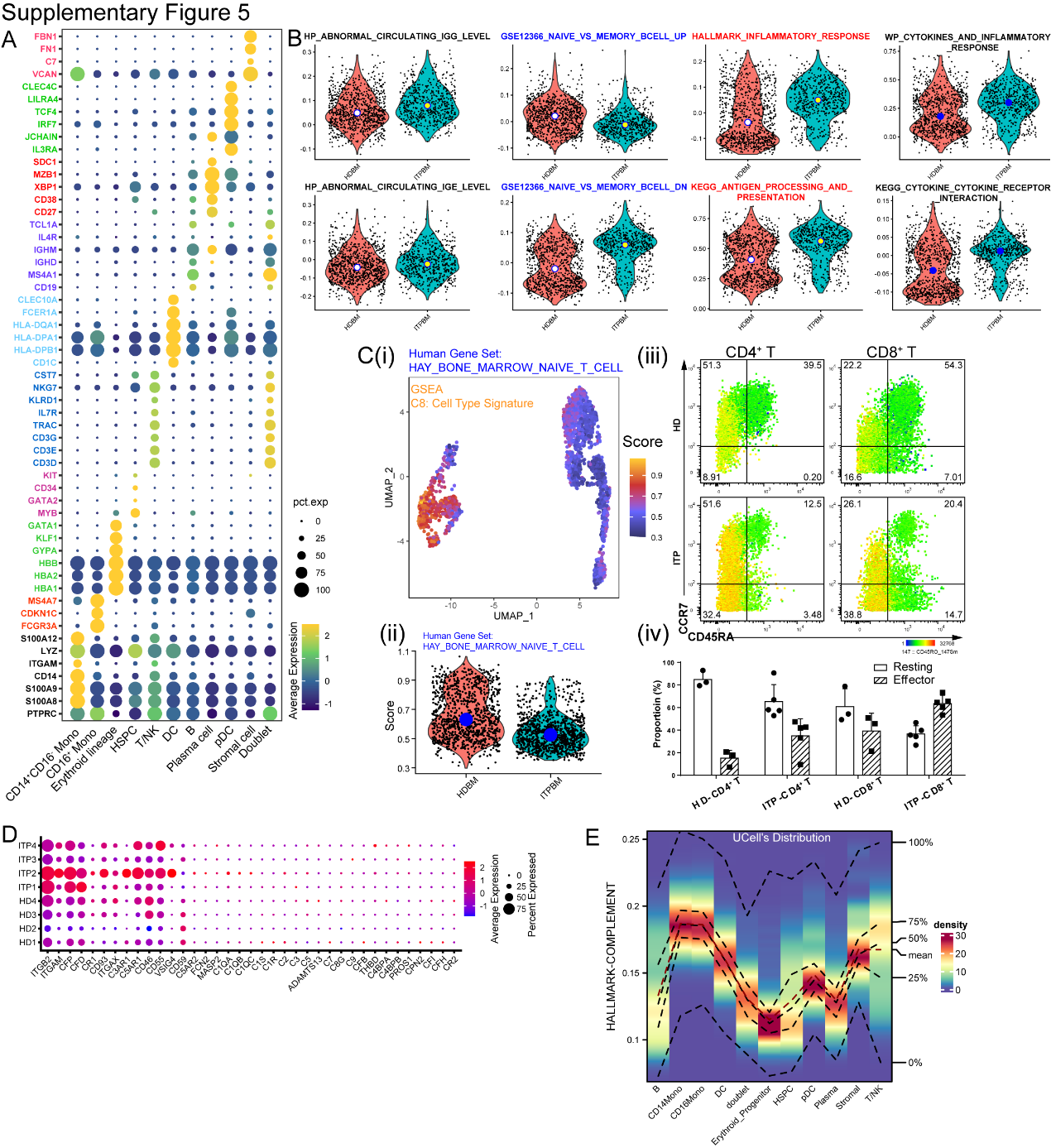


**Figure S5. Single-cell transcriptomics dissects the transcriptome alterations of immune and hematopoietic cells in ITP.**

**(A).** Bubble plot displaying representative lineage-specific signatures used to discriminate between the 11 subclusters.

**(B).** Violin plot of B and plasma cells showing the scores of “HP_ABNORMAL_CIRCULATING_IGG_LEVEL,” “HP_ABNORMAL_CIRCULATING_IGE_LEVEL G,” “GSE12366_NAIVE_VS_MEMORY_BCELL_UP,” “GSE12366_NAIVE_VS_MEMORY_BCELL_DN,” “HALLMARK_INFLAMMATORY_RESPONSE,” “KEGG_ANTIGEN_PROCESSING_AND_PRESENTATION,” “WP_CYTOKINES_AND_INFLAMMATORY_RESPONSE,” and “KEGG_CYTOKINE_CYTOKINE_RECEPTOR_INTERACTION” between ITP and HD.

**(C).** **(i)** UMAP of T cells showing the scores of “HAY_BONE_MARROW_NAIVE_T_CELL”. **(ii)** Violin plot of T cells showing the scores of “HAY_BONE_MARROW_NAIVE_T_CELL” between ITP and HD Representative mass cytometry analysis of the surface markers CCR7 and CD45RA in BM T cells from HDs and patients with ITP. **(iii)** Dot plot coloring is based on the expression level of CD45RO, blue indicates the lowest expression, and red indicates the highest expression. **(iv)** Column charts show the proportions of the representative subsets. Resting subset was defined as CCR7^+^ population, and effector subset was defined as CCR7^-^ population.

**(D).** Bubble plot of complement pathway associated gene expression in BMMC from patients with ITP versus HDs.

**(E).** Distribution of the scores of “UCell: HALLMARK-COMPLEMENT” in various subsets.


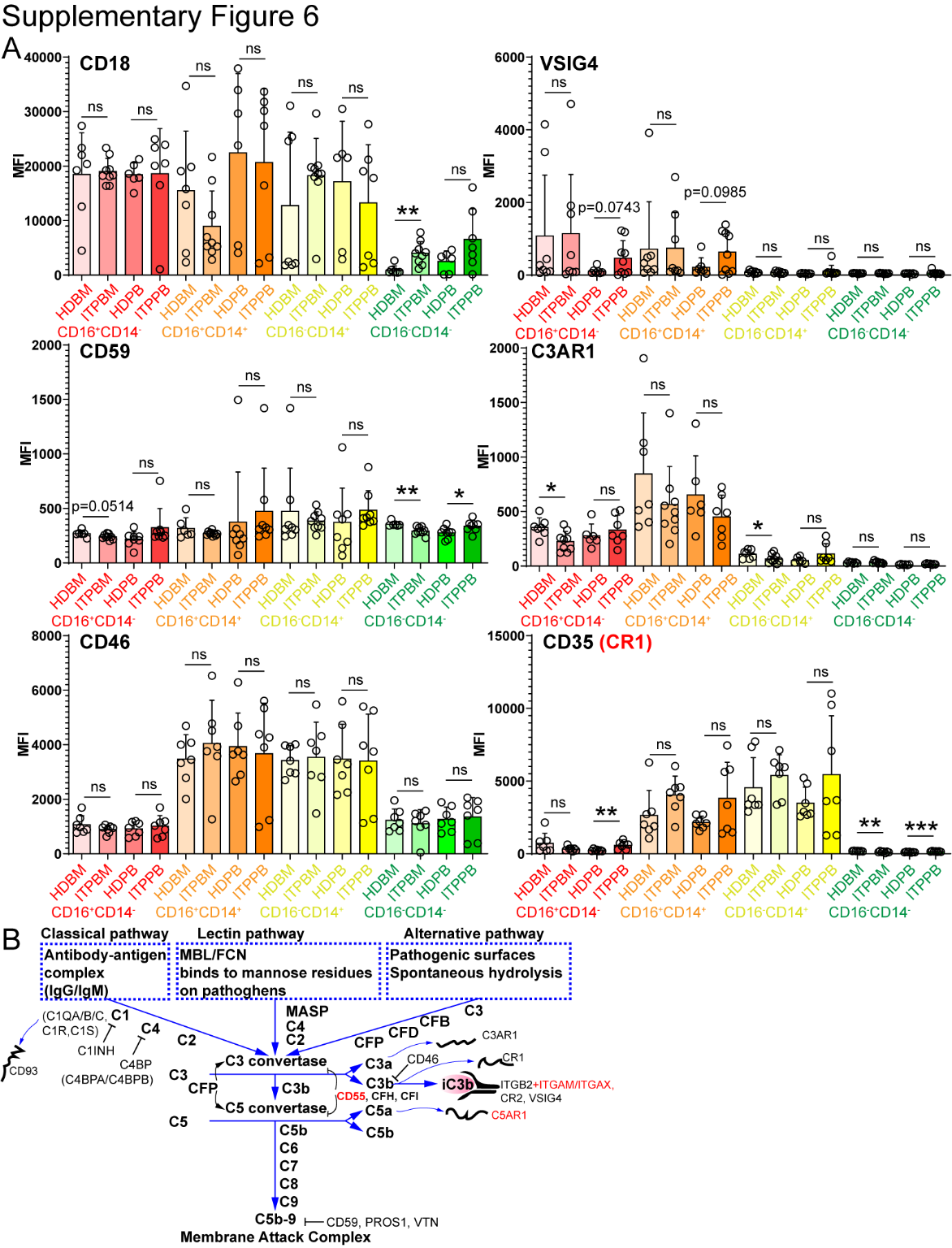


**Figure S6. The differences of complement-associated surface proteins in HD and patients with ITP.**

**(A).** Summary graphs showing the median fluorescent intensity (MFI) of CD59, CD18, CD46, VSIG4, C3AR1, and CD35 in different subsets of BMMC and PBMC from patients with ITP and healthy donors. P value significance represented by *, <0.05; **, <0.01; ***, <0.001; ****, <0.0001.

**(B).** Schematic overview of the complement system and the interaction of its derivatives with the receptor. Significant proteins involved in the abnormal complement-associated immune regulation are shown in red.


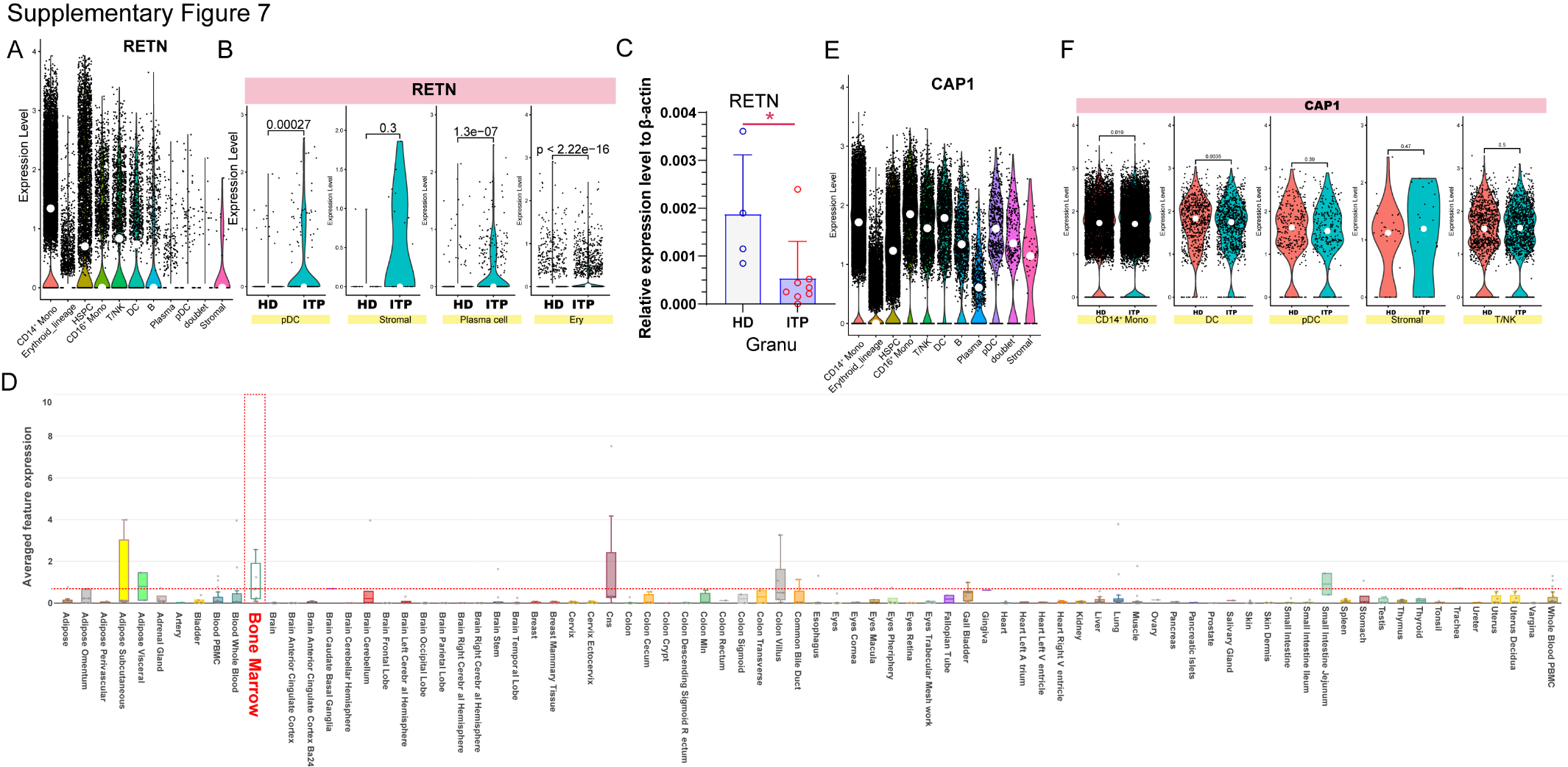


**Figure S7. Resistin was widely expressed in various immune and hematopoietic subsets enhancing the inflammatory responses.**

**(A).** Violin plots illustrating the distribution of *RETN* expression across various cell types.

**(B).** Violin plots illustrating the expression distribution of *MIF* in pDC, stromal cells, plasma cells, and erythroid lineage across various groups. P value significance represented by *, <0.05; **, <0.01; ***, <0.001; ****, <0.0001.

**(C).** Quantitative *RETN* transcripts in BM granulocytes from HDs and ITP patients by using qPCR. *P* value significance represented by *, <0.05; **, <0.01; ***, <0.001; ****, <0.0001.

**(D).** Box plot showing the *RETN* expression levels across various human tissue types and organs (https://www.singlecellatlas.org/gene-search); BM is highlighted.

**(E).** Violin plots illustrating the distribution of *CAP1* expression across various cell types.

**(F).** Violin plots illustrating the distribution of *CAP1* expression across various cell types, including CD14^+^ monocytes, DC, pDC, stromal cells, and T/NK cells, in HD and patients with ITP. P value significance represented by *, <0.05; **, <0.01; ***, <0.001; ****, <0.0001.


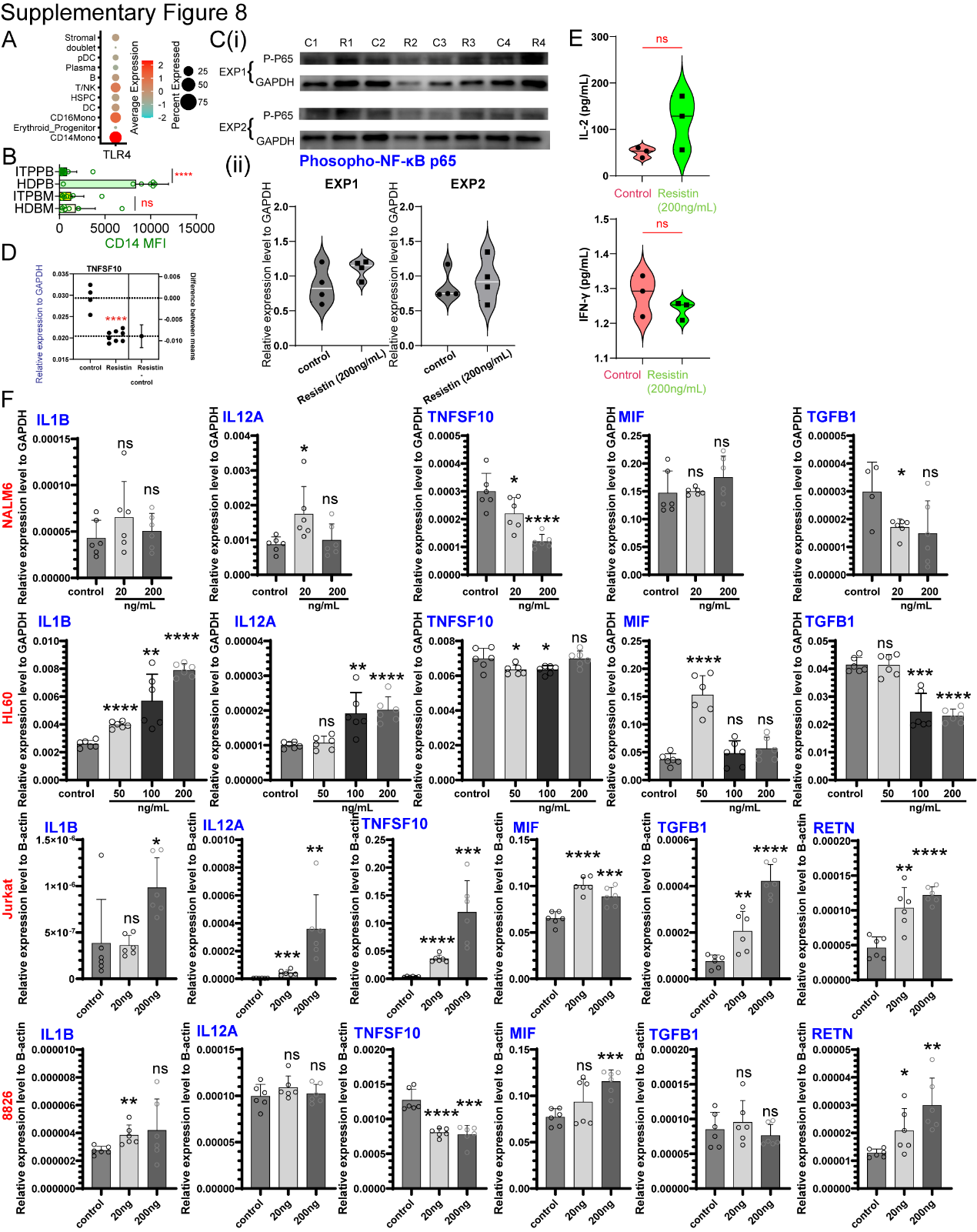


**Figure S8. Resistin is a contributor of BM inflammatory environment.**

**(A).** Bubble plot of TLR4 expression across various cell types from patients with ITP and HDs.

**(B).** Summary graphs showing the median fluorescent intensity (MFI) of TLR4 in different subsets of BMMC and PBMC from patients with ITP and HDs. *P* value significance represented by *, <0.05; **, <0.01; ***, <0.001; ****, <0.0001.

**(C).** Western blot analysis of P-P65 and GAPDH protein levels in THP-1 cells after stimulation with vehicle or resistin (i). Histograms showing the quantification of P-P65 protein (ii). This experiment was conducted by another researcher to verify independent repeatability.

**(D).** qPCR validation of relative gene expression for *TNFSF10* in THP-1 cells after resistin stimulation. *P* value significance represented by *, <0.05; **, <0.01; ***, <0.001; ****, <0.0001.

**(E).** Levels of IL-2 and IFN-γ in supernatants of THP-1 cultures, measured using ELISA after treatment with resistin. *P* value significance represented by *, <0.05; **, <0.01; ***, <0.001; ****, <0.0001.

**(F).** qPCR validation of the relative expression of selected genes (*IL1B*, *IL12A*, *TNFSF10*, *MIF*, *TGFB1*, and *RETN*) in various cell lines after resistin stimulation. *P* value significance represented by *, <0.05; **, <0.01; ***, <0.001; ****, <0.0001.


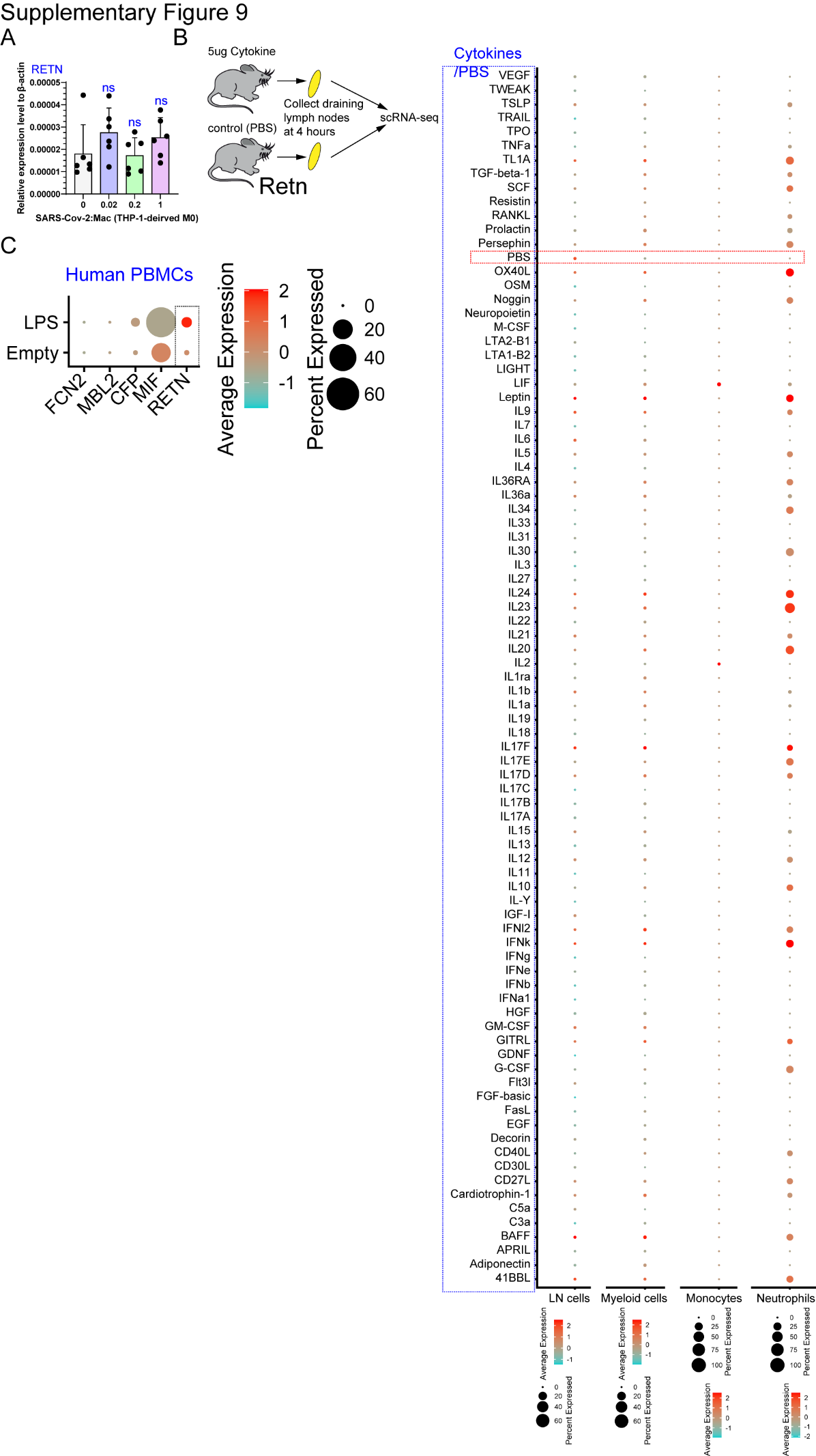


**Figure S9. RETN expression is induced by LPS, virus, and various cytokines.**

**(A).** Quantification of *RETN* mRNA expression in control and pseudovirus-treated groups. THP-1 cells were differentiated into the M0 state and then stimulated with different concentrations of the pseudovirus.

**(B).** Bubble plot of *RETN* expression in all lymph node cells, myeloid subset, monocyte subset, and neutrophil subset from the lymph nodes of mice treated with different cytokines and PBS. This data was acquired from “Immune Dictionary (<https://www.immune-dictionary.org/app/home>),” and analyzed utilizing Seurat.

**(C).** Bubble plot of selected genes (*FCN2*, *MBL2*, *CFP*, *MIF*, and *RETN*) in human PBMCs treated with LPS versus vehicle. scRNA-seq datasets of LPS-treated PBMCs and their control groups were obtained from EMBL-EBI (https://www.ebi.ac.uk/ena/browser/view/PRJEB40448), and analyzed using Seurat.


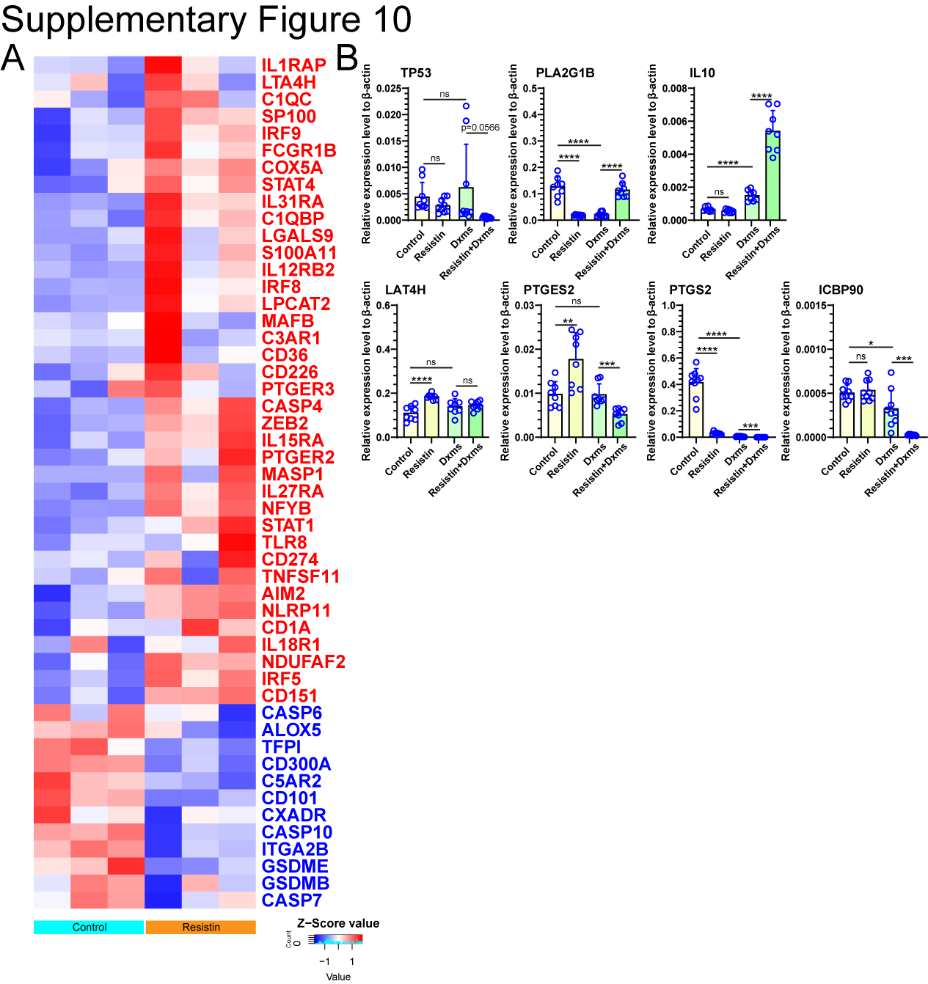


**Figure S10. Resistin is a driver of Dxms resistance.**

**(A).** Heatmap showing the relative expression levels of selected genes in THP-1 cells treated with MIF (n=3) or without resistin (n=3).

**(B).** *TP53*, *PLA2*, *IL10*, *LAT4H*, *PTGES2*, *PTGS2*, *ALOX5*, and *ICBP90* transcript levels were estimated after resistin and/or dxms stimulation in the THP-1 cell line. *P* value significance represented by *, <0.05; **, <0.01; ***, <0.001; ****, <0.0001.


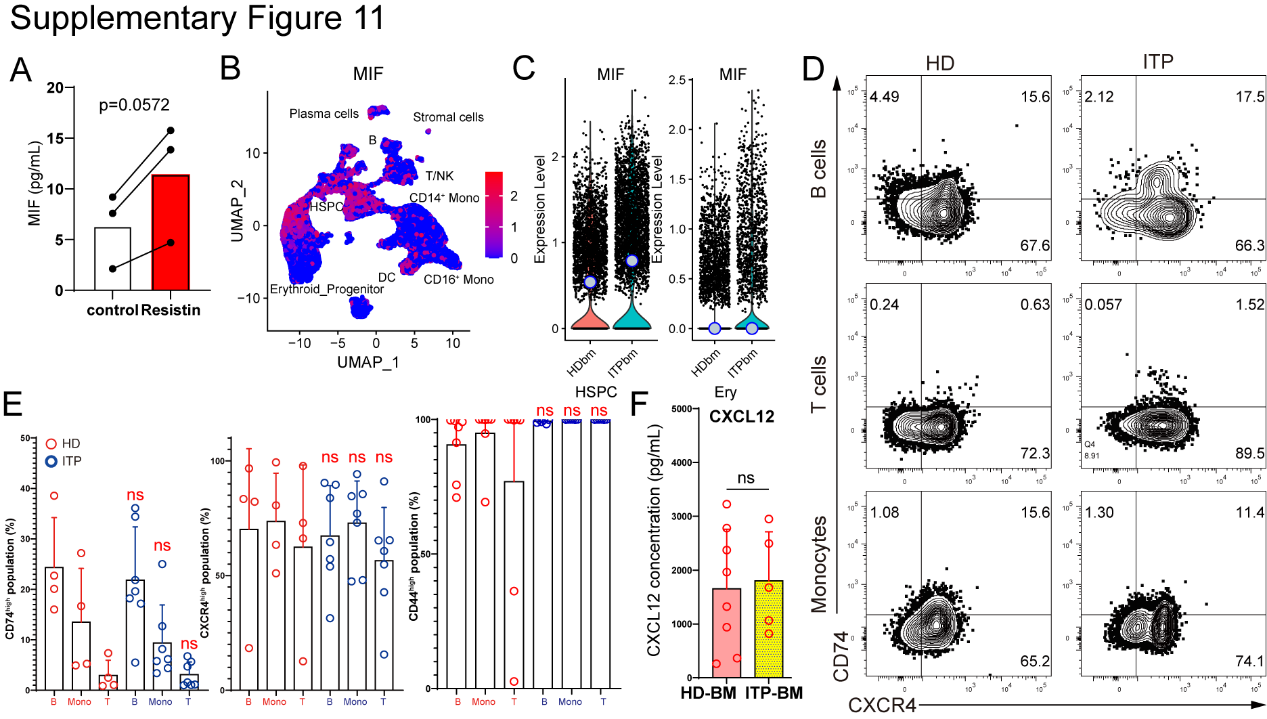


**Figure S11. Resistin upregulates MIF promoting further amplified autoimmune responses.**

**(A).** Levels of MIF in the supernatants of CD19^+^ B cell cultures were measured using ELISA after treatment with resistin.

**(B).** Feature plot of MIF in the UMAP of all BMMCs, including ITP patients and HDs.

**(C).** Violin plots illustrating the expression distribution of *MIF* in HSPCs and erythroid lineages across the various groups.

**(D).** The proportions of CD74^+^ and CXCR4^+^ cells among B cells, T cells, and monocytes were determined using FCM.

**(E).** Summary graphs showing the percentages of CD74^+^, CD44^+^, and CXCR4^+^ cells in B cells, T cells, and monocytes of BMMC from patients with ITP and HDs.

**(F).** Levels of CXCL12 in BM plasma samples from patients with ITP (n=8) and HDs (n=5) measured using ELISA.


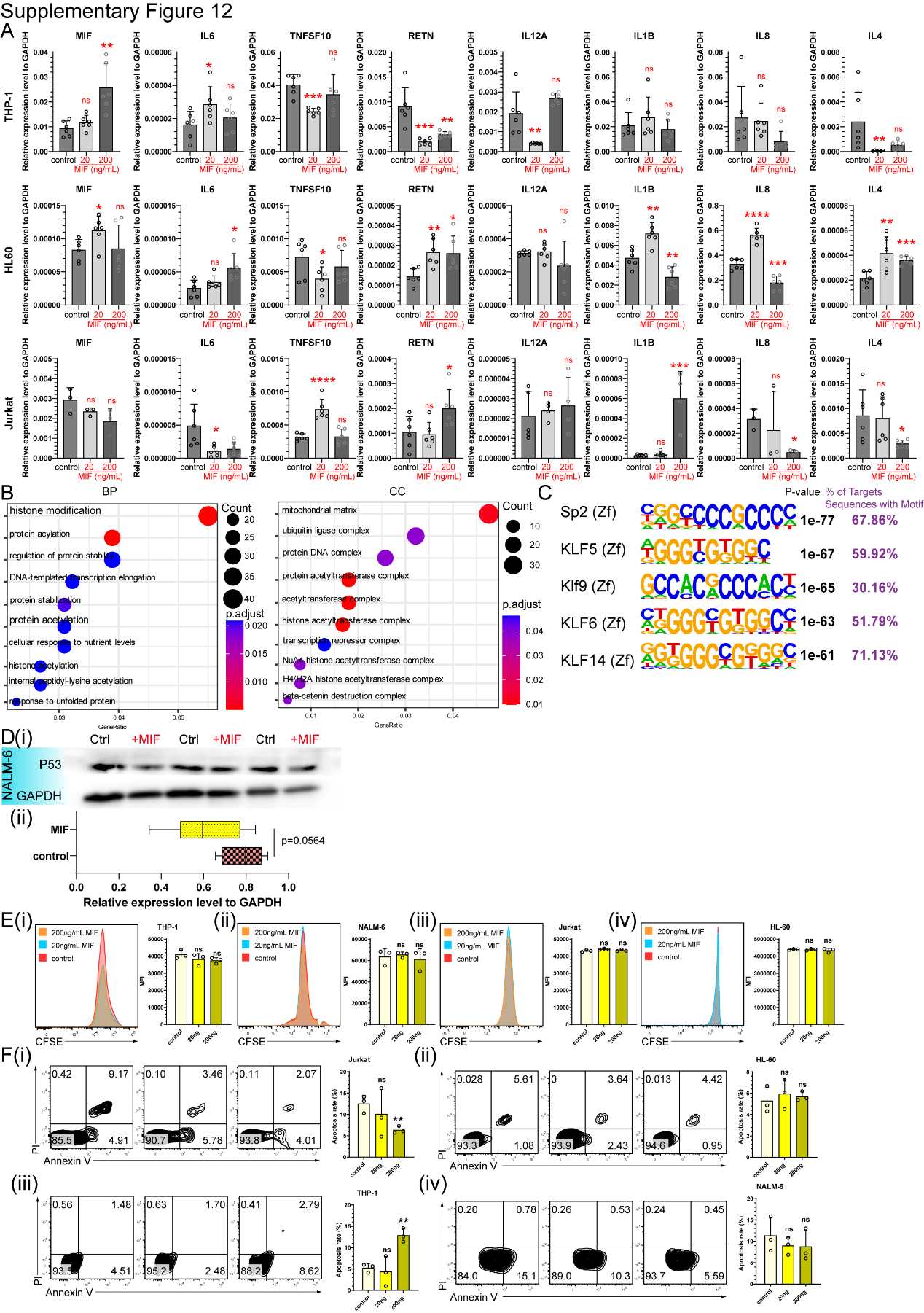


**Figure S12. MIF alters the immune functions via reprogramming the cell fates of immune cells.**

**(A).** qPCR validation of the relative expression of selected genes in various cell lines after MIF stimulation. *P* value significance represented by *, <0.05; **, <0.01; ***, <0.001; ****, <0.0001.

**(B).** GO enrichment analysis of DEGs identified using ATAC-seq analysis.

**(C).** MIF-treatment enriched motifs containing *Sp2*, *KLF5*, *Klf9*, *KLF6*, and *KLF14*.

**(D).** Western blot analysis of TP53 protein levels in NALM6 cells after stimulation with vehicle or MIF.

**(E).** Representative flow cytometry plots showing CFSE intensity of THP-1 **(i)**, NALM6 **(ii)**, Jurkat **(iii)**, and HL60 **(iv)** with MIF treatment after 24 h. *P* value significance represented by *, <0.05; **, <0.01; ***, <0.001; ****, <0.0001.

**(F).** Representative flow cytometry plots showing proportions of apoptotic cells in Jurkat **(i)**, HL-60 **(ii)**, THP-1 **(iii)**, and, NALM6 **(iv)** treated with MIF after 24 h. *P* value significance represented by *, <0.05; **, <0.01; ***, <0.001; ****, <0.0001.


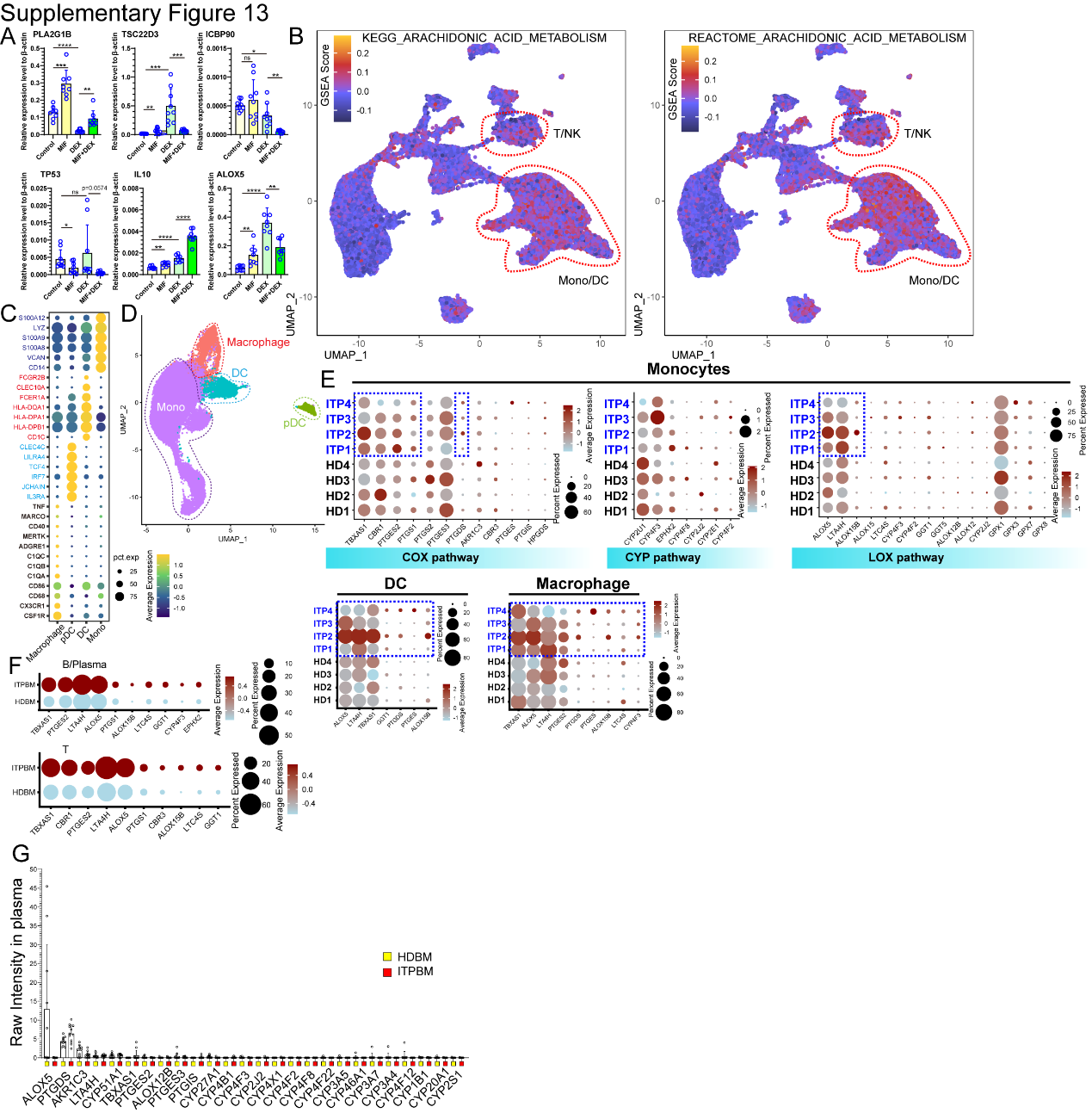


**Figure S13. Oxidized fatty acid metabolism was altered in patients with ITP.**

**(A).** qPCR analysis of selected genes in THP-1 cells after stimulation with vehicle, dxms, MIF, or a combination of MIF and dxms. *P* value significance represented by *, <0.05; **, <0.01; ***, <0.001; ****, <0.0001.

**(B).** UMAP of BM mononuclear cells showing the scores of “KEGG_ARACHIDONIC_ACID_METABOLISM” and “REACTOME_ARACHIDONIC_ACID_METABOLISM.”

**(C).** Bubble plot displaying representative lineage-specific signatures used to discriminate the four subclusters (macrophages, pDC, DC, and monocytes).

**(D).** UMAP visualization of single-cell transcriptional states obtained from pDC and myeloid cells of patients with ITP compared with healthy donors. Distinct colors represent individual cell types determined based on gene expression; pDC, plasmacytoid dendritic cells; DC, dendritic cells; Mono, monocytes.

**(E).** Bubble plot of oxidized fatty acid metabolism-associated gene expression in different subsets (monocytes, DC, and macrophages) from patients with ITP versus HDs.

**(F).** Bubble plot of oxidized fatty acid metabolism-associated gene expression in different subsets (B/plasma cells and T cells) of patients with ITP versus HDs.

**(G).** Abundance differences in oxidized fatty acid metabolism-associated proteins between ITP patients BM plasma samples and healthy donor BM plasma samples assessed by proteomic profiling. Data represent the mean or the mean with SD.

**Table S1. Information of patients with ITP and healthy donors for scRNA-seq analysis**

| **NO.** | **Sample NO.** | **Age (year)** | **gender** | **Disease classification** | **Interval from onset to diagnosis (m)** | **Peripheral blood count** | | | | | **Bone marrow hypoplasia** | **Cytogenetic** | **Genetic mutation** | **Description** |
| --- | --- | --- | --- | --- | --- | --- | --- | --- | --- | --- | --- | --- | --- | --- |
|  |  |  |  |  |  | **WBC(*10e9/L)** | **ANC(*10e9/L)** | **HGB(g/L)** | **PLT(*10e9/L)** | **ARC(*10e9/L)** |  |  |  |  |
| **HD1** | **HDBM1** | **41** | **M** | **--** | **--** | **8.12** | **4.16** | **133** | **271** | **--** | **--** | **--** | **--** | **BMMC of Healthy donor 1#** |
| **HD2** | **HDBM2** | **35** | **M** | **--** | **--** | **10.85** | **5.74** | **157** | **272** | **61** | **--** | **--** | **--** | **BMMC of Healthy donor 2#** |
| **HD3** | **HDBM3** | **35** | **F** | **--** | **--** | **6** | **3.37** | **100** | **264** | **71.5** | **--** | **--** | **--** | **BMMC of Healthy donor 3#** |
| **HD4** | **HDBM4** | **16** | **M** | **--** | **--** | **6.47** | **3.29** | **152** | **274** | **--** | **--** | **--** | **--** | **BMMC of Healthy donor 4#** |
| **ITP1** | **ITPBM1** | **59** | **M** | **ITP** | **36** | **7.69** | **6.31** | **148** | **39** | **84.2** | **Normal** | **Normal** | **--** | **BMMC of patient with ITP (1#)** |
| **ITP2** | **ITPBM2** | **51** | **F** | **ITP** | **0.1** | **5.44** | **3.76** | **128** | **14** | **70.9** | **Normal** | **Normal** | **--** | **BMMC of patient with ITP (2#)** |
| **ITP4** | **ITPBM3** | **35** | **F** | **ITP** | **60** | **7.93** | **5.21** | **128** | **34** | **69.8** | **Normal** | **Normal** | **--** | **BMMC of patient with ITP (3#)** |
| **ITP5** | **ITPBM4** | **71** | **F** | **ITP** | **48** | **3.73** | **2.64** | **117** | **2** | **93.6** | **Normal** | **Normal** | **--** | **BMMC of patient with ITP (4#)** |

**Table S2.** qPCR primers

| **Gene** | **Primer** | **Sequences** |
| --- | --- | --- |
| Human GAPDH | Forward | ATCAATGGAAATCCCATCACCA |
|  | Reverse | GACTCCACGACGTACTCAGCG |
| Human β-actin (ACTB) | Forward | CCTGGCACCCAGCACAAT |
|  | Reverse | GGGCCGGACTCGTCATAC |
| Human MBL2 | Forward | AAAAAGTCCGGATGGTGATAGT |
|  | Reverse | CCACTTTTTGATACGTGCCATT |
| Human FCN2 | Forward | CAACTACCAGTTTGCTAAGTACAG |
|  | Reverse | GGTCTTTGGTGGAGAAGGACTG |
| Human CFP | Forward | GAATGGGCAGTGCTCTGGAAAG |
|  | Reverse | TTGGAGCAGGTGACAGAGCAAG |
| Human MIF | Forward | CATCGTAAACACCAACGTGC |
|  | Reverse | CCGCGTTCATGTCGTAATAG |
| Human RETN | Forward | TAGGGCAATAAGCAGCATTG |
|  | Reverse | ACTGGCAGTGACATGTGGTC |
| Human PLA2G1B | Forward | ACAACTACGGCTGCTACTGTGG |
|  | Reverse | GTGTACGGGTTGTCCAGCAGAA |
| Human ANXA1 | Forward | GCGAAACAATGCACAGCGTCAAC |
|  | Reverse | CAACCTCCTCAAGGTGACCTGT |
| Human TSC22D3 | Forward | GCGTGAGAACACCCTGTTGA |
|  | Reverse | TCAGACAGGACTGGAACTTCTCC |
| Human PTGS2 | Forward | CTGGCGCTCAGCCATACAG |
|  | Reverse | CGCACTTATACTGGTCAAATCCC |
| Human ICBP90 | Forward | GTAAAGTGGAGGAGACGTTCC |
|  | Reverse | TTGCTGCCACCAACTCTTTA |
| Human TP53 | Forward | CAGCACATGACGGAGGTTGT |
|  | Reverse | TCATCCAAATACTCCACACGC |
| Human ALOX5 | Forward | ACAAGCCCTTCTACAACGACT |
|  | Reverse | AGCTGGATCTCGCCCAGTT |
| Human PTGES2 | Forward | CTGGCGTCCTTTGACTACATT |
|  | Reverse | TCTTGAGTCGCTTGCTGATG |
| Mouse Mbl2 | Forward | TGACAGTGGTTTATGCAGAGAC |
|  | Reverse | CGTCACGTCCATCTTTGCC |
| Mouse Cfp | Forward | CTTTTGTGCTGGTGATGCCACTC |
|  | Reverse | CAGTTGATGGACATTCTCAGCCG |
| Mouse Fcna | Forward | CACCATCCATCTTCCTGACTGC |
|  | Reverse | ATAGGAGTCCCAGTCTCGGAAG |
| Mouse Fcnb | Forward | TCTTCCAGAGGAGGCTTGAC |
|  | Reverse | GGGTGGTTAGAGCGTGGATA |
| Mouse Mif | Forward | GAACCGCAACTACAGTAAGCTGC |
|  | Reverse | ACGTTGGCAGCGTTCATGTCGT |
| Mouse Ptgs1 | Forward | GAATGCCACCTTCATCCGAGAAG |
|  | Reverse | GCTCACATTGGAGAAGGACTCC |
| Mouse Ptgs2 | Forward | GCGACATACTCAAGCAGGAGCA |
|  | Reverse | AGTGGTAACCGCTCAGGTGTTG |
| Mouse Tsc22d3 | Forward | TCAATGAGGGCATCTGCAACCG |
|  | Reverse | CATCAGGTGGTTCTTCACGAGG |
| Mouse Slirp | Forward | CAGAAAAATTCCTTGGACCGCGG |
|  | Reverse | AACCCATGCCTCTGTGAAAGCC |
| Mouse Ephx2 | Forward | CCCTCAAGCAGTGTTCATTGGC |
|  | Reverse | ATCTGGTGGCATAAACGGCGTG |
| Mouse Pf4 | Forward | GTTGTTTCTGCCAGCGGTGGTT |
|  | Reverse | ACAGTGGCGTCCTGCCTTGATC |
| Mouse Hif1a | Forward | CCTGCACTGAATCAAGAGGTTGC |
|  | Reverse | CCATCAGAAGGACTTGCTGGCT |
| Mouse Retn | Forward | CATGCCACTGTGTCCCATCGAT |
|  | Reverse | ACTTCCCTCTGGAGGAGACTGT |
| Mouse Il1b | Forward | TGGACCTTCCAGGATGAGGACA |
|  | Reverse | GTTCATCTCGGAGCCTGTAGTG |

1. Jones RC, Karkanias J, Krasnow MA, et al. The Tabula Sapiens: A multiple-organ, single-cell transcriptomic atlas of humans. *Science (New York, NY)*. May 13 2022;376(6594):eabl4896. doi:10.1126/science.abl4896

2. Xu J, Guo P, Hao S, et al. A spatiotemporal atlas of mouse liver homeostasis and regeneration. *Nature Genetics*. 2024/04/16 2024;doi:10.1038/s41588-024-01709-7
